# Phylogenetic diversity of putative nickel-containing carbon monoxide dehydrogenase-encoding prokaryotes in the human gut microbiome

**DOI:** 10.1099/mgen.0.001285

**Published:** 2024-08-21

**Authors:** Yuka Adachi Katayama, Ryoma Kamikawa, Takashi Yoshida

**Affiliations:** 1 Graduate School of Agriculture, Kyoto University, Kitashirakawa Oiwake-cho, Sakyo-ku, Kyoto 606-8502, Japan

**Keywords:** *Blautia*, carbon monoxide-utilizing prokaryotes, human gut microbiome, nickel-containing carbon monoxide dehydrogenase, WLP

## Abstract

Although the production of carbon monoxide (CO) within the human body has been detected, only two CO-utilizing prokaryotes (CO utilizers) have been reported in the human gut. Therefore, the phylogenetic diversity of the human gut CO-utilizing prokaryotes remains unclear. Here, we unveiled more than a thousand representative genomes containing genes for putative nickel-containing CO dehydrogenase (pCODH), an essential enzyme for CO utilization. The taxonomy of genomes encoding pCODH was expanded to include 8 phyla, comprising 82 genera and 248 species. In contrast, putative molybdenum-containing CODH genes were not detected in the human gut microbial genomes. pCODH transcripts were detected in 97.3 % (*n*=110) of public metatranscriptome datasets derived from healthy human faeces, suggesting the ubiquitous presence of prokaryotes bearing transcriptionally active pCODH genes in the human gut. More than half of the pCODH-encoding genomes contain a set of genes for the autotrophic Wood–Ljungdahl pathway (WLP). However, 79 % of these genomes commonly lack a key gene for the WLP, which encodes the enzyme that synthesizes formate from CO_2_, suggesting that potential human gut CO-utilizing prokaryotes share a degenerated gene set for WLP. In the other half of the pCODH-encoding genomes, seven genes, including putative genes for flavin adenine dinucleotide-dependent NAD(P) oxidoreductase (FNOR), ABC transporter and Fe-hydrogenase, were found adjacent to the pCODH gene. None of the putative genes associated with CO-oxidizing respiratory machinery, such as energy-converting hydrogenase genes, were found in pCODH-encoding genomes. This suggests that the human gut CO utilization is not for CO removal, but potentially for fixation and/or biosynthesis, consistent with the harmless yet continuous production of CO in the human gut. Our findings reveal the diversity and distribution of prokaryotes with pCODH in the human gut microbiome, suggesting their potential contribution to microbial ecosystems in human gut environments.

**Impact Statement**

Carbon monoxide (CO) is a ubiquitous gas that can be utilized as energy and carbon sources for certain micro-organisms containing CO dehydrogenase (Ni-CODH). CO is produced via multiple routes, such as haem degradation, even in the human body, including the intestinal cells and human gut microbes. Nevertheless, only a limited number of CO utilizers in the human gut microbiome have been reported. Our study revealed that a significant proportion of human gut microbial genomes belonging to diverse phyla possess genes for putative anaerobic Ni-CODH (pCODH), and they distribute and transcribe those genes prevalently in the human gut microbiome. Based solely on predictions from genetic arrangements, none of the pCODHs in the human gut microbiome were associated with CO oxidation, typically labelled as CO-detoxifying functions in the environment. Nearly half of the pCODH-encoding genomes in the human gut contained putative genes for the Wood–Ljungdahl pathway (WLP), one of the most well-known autotrophic pathways. However, the majority of these genomes have potentially remodelled the gene set for the WLP to the degenerated, heterotrophic form. Moreover, CO-utilizing functions other than WLP were predicted to be present in nearly half of the pCODH-encoding genomes. Our findings would provide informatic bases to deepen our understanding of the diversity of prokaryotic species with the genomes encoding pCODH that are prevalently found in the human gut microbiome.

## Data Summary

The human gut prokaryotic genomes were downloaded from the HumGut database (https://arken.nmbu.no/~larssn/humgut/) [1]. The accession numbers of CODH/ACS-encoding genomes from environments without host association [2] are listed in Table S1. Metatranscriptomic datasets were downloaded from the National Center for Biotechnology Information (NCBI) Sequence Read Archive (SRA) under the BioProject accession numbers PRJNA354235 and PRJNA707065, and their accession IDs are listed in Table S2.

## Introduction

Carbon monoxide (CO) is a colourless, odourless and tasteless gas that is produced via the combustion of organic compounds and cellular metabolism [3]. While CO is widely labelled as a toxic molecule due to its high affinity for haemoproteins, causing severe effects on human health at exposure to >100 ppm [3,5], the human body constitutively produces 0.5–4.5 ppm of CO per hour on average, primarily through haem degradation using haem oxygenase 1 and 2 (HO-1 and HO-2) [6,8]. In healthy human gut cells, HO-1 is expressed in response to injury and/or inflammation, while HO-2 is constitutively expressed, producing one molecule of CO per one molecule of haem degraded [9]. In addition to the human host, gastrointestinal microbes also contribute to CO production using HOs and other metabolic pathways [8]. At low concentrations, CO has shown beneficial effects on human health as an anti-inflammatory agent and a neurotransmitter [8,9]. From the human body and exogenous sources, CO is assumed to accumulate in the gastrointestinal tract, potentially influencing the composition of intestinal microbiota [8].

Prokaryotes utilizing CO are called CO utilizers and have been found in various environments, including soil [10], lake [11], freshwater lake sediments [12], bioreactors [13], hot springs [14], deep hydrothermal vents [15] and even gut environment [16]. All CO utilizers possess CO dehydrogenase (CODH), which catalyses the reversible conversion of CO_2_ and CO (CO_2_ + 2H^+^ + 2e^−^ <=> CO + H_2_O) [17,18]. CODHs are physiologically and structurally diverse enzymes that form two major groups, namely, nickel- and molybdenum-containing CODHs (Ni-CODH and Mo-CODH), of which Ni-CODH works under anaerobic conditions [19]. The catalytic subunit of Ni-CODH (CooS and CdhA) is phylogenetically and structurally classified into eight distinct clades, A to H, and mini-CODH [2,20]. Through reactions catalysed by these CODHs, CO utilizers utilize CO as a carbon source and/or as the low redox potential (*E*^0^′= −520 mV) for carbon fixation and/or energy conservation in various CO-metabolizing pathways and respiratory chains. In energy conservation, the reducing power of CO is supplied to various terminal electron acceptors, including proton, thiosulphate and nitrate, in the electron transport chains to generate an electrochemical gradient for ATP synthesis [17,21,23]. In addition, through the reduction and oxidation of the ferredoxin (Fd)-like protein CooF and flavin adenine dinucleotide-dependent NAD(P) oxidoreductase (FNOR), the reducing power of CO is supplied to Fd_ox_ and NAD^+^, thereby generating Fd_red_ and NADH, which is available for various respiration and biosynthesis reactions [20,24, 25]. Although CO-oxidizing machinery, such as hydrogenogenic CO oxidation using Ni-CODH/energy-converting hydrogenase (Ech), are not essential for many bacteria, CO oxidation contributes to auxiliary energy gain, thereby enhancing bacterial survival [21,26]. In addition, due to the nearly diffusion-limited rate of CO oxidation, CO oxidizers have been suggested to play a role for CO detoxification in microenvironments [27,29].

As the CO-mediated CO_2_ fixation pathways, the Wood–Ljungdahl pathway [WLP; reductive acetyl-coenzyme A (CoA) pathway] has been best studied. The WLP is recognized as the most ancient autotrophic pathway, which was possessed by the last universal common ancestor [30,32]. This pathway consists of two branches, carbonyl and methyl branches [32,33]. In the carbonyl branch, CO_2_ is reduced to CO by Ni-CODH and incorporated into the carbonyl moiety of acetyl-CoA. Alternatively, CO can be obtained directly from the surrounding environment and incorporated into the carbonyl branch [33]. In the methyl branch, formate is synthesized from CO_2_ by formate dehydrogenase (Fdh), which is then reduced stepwise to a methyl moiety (CH_3_-) by formyl-tetrahydrofolate synthase (Fhs), methylene-tetrahydrofolate dehydrogenase/cyclohydrolase (FolD) and methylene-tetrahydrofolate reductase (MetF). The generated carbonyl and methyl moieties are incorporated into acetyl-CoA along with CoA by acetyl-CoA synthase (ACS), which forms a complex with Ni-CODH (CODH/ACS). Acetyl-CoA is then converted into various compounds such as short-chain fatty acids, including acetate, by CO utilizers [16,34]. The WLP has been shown to provide remarkable metabolic versatility to WLP-bearing prokaryotes. For instance, the WLP serves as an electron sink during the mixotrophic growth by accepting electrons provided via reactions by glycolysis and/or pyruvate: Fd oxidoreductase (PFOR; pyruvate+CoA+2 Fd_ox_ <=> acetyl CoA + CO_2_ + Fd_red_) [19,30, 35]. In addition, WLP is linked to energy conservation using Rnf or Ech complexes, where the oxidation of Fd_red_ is coupled with the reduction of NAD^+^ and protons, respectively [36,37]. MetF in the methyl branch is also linked to the generation of proton gradient in some acetogens, such as *Sporomusa ovata* [38]. The genes for the CO-associated enzymes, including FNOR and ACS, tended to be located in close proximity to Ni-CODH genes in the genomes of CO utilizers, and thus, functions of Ni-CODHs have been predicted based on their genomic context [2,20, 39, 40].

Although the CO-utilizing abilities of human gut prokaryotes have not yet been fully elucidated, accumulating evidence suggests that CO utilizers are present in the human gut microbiome. For instance, the previous study demonstrated the rapid consumption of CO by fresh human faecal samples [41]. The two human gut acetogenic bacteria, *B. luti* and *B. wexlerae*, were found to consume CO via the Fdh-lacking WLP when formate was added to the medium [16]. Furthermore, several gastrointestinal prokaryotes such as *Clostridium*, *Clostridioides* and *Marvinbryantia* possess functional WLPs [35,42, 43]. However, previously reported CO-utilizing pathway in the human gut is limited to the WLP, and the diversity of CO utilizers and the presence of other CO-utilizing/oxidizing functions have not fully been elucidated. In this study, we performed a deep bioinformatic-based analysis to gain insight into the phylogenetic diversity of the human gut microbial genomes encoding putative Ni-CODH, their estimated metabolic pathways associated with CO utilization and their distribution in the human gut microbiome.

## Methods

### Genome sources

**Fig. 1.**
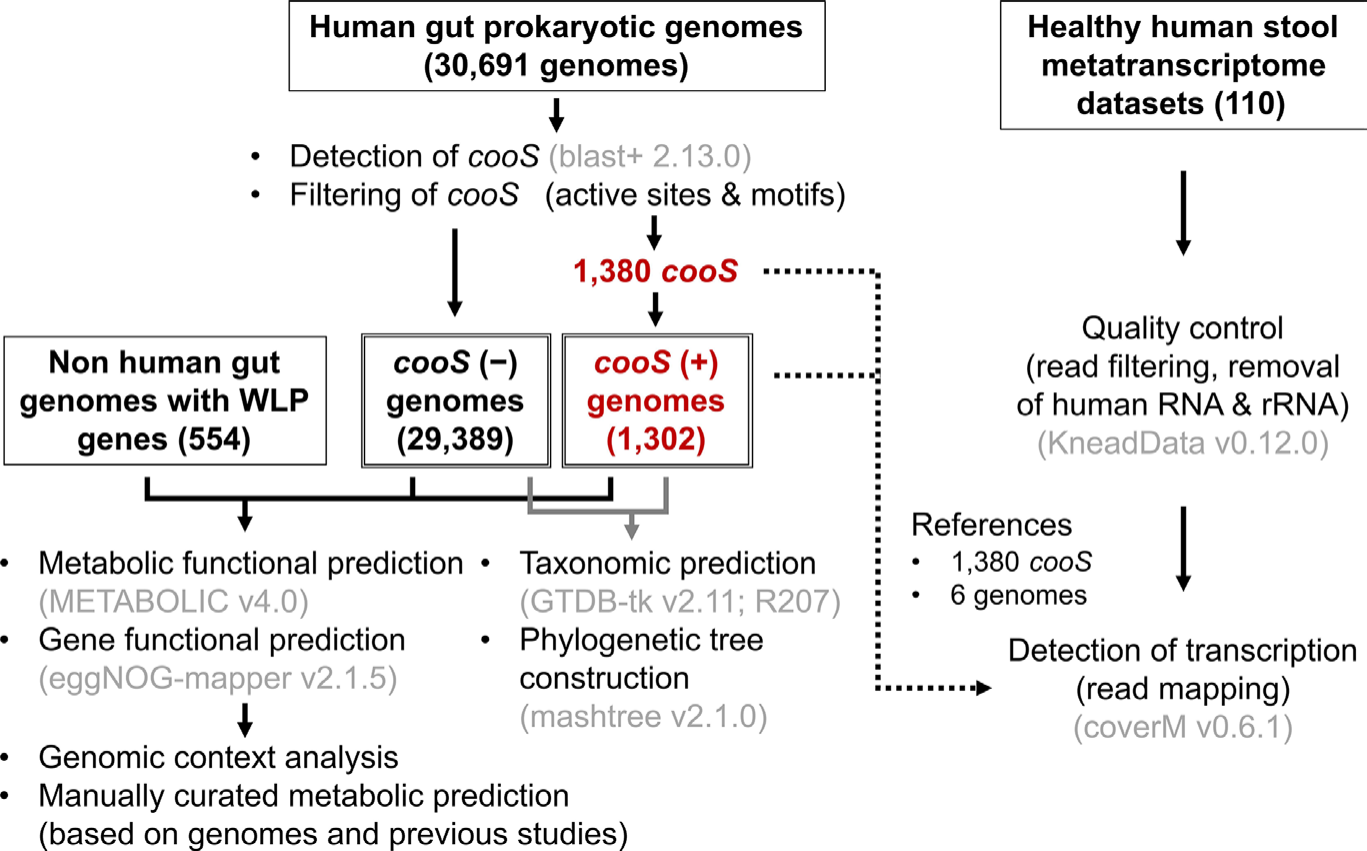
Workflow of this study. The study utilized publicly available human gut prokaryotic genomes from the HumGut database (30 691 genomes) and metatranscriptomic datasets from 110 healthy individuals. The putative Ni-CODH (pCODH)-encoding genomes were identified using *cooS* as genetic markers, and sequences were filtered based on active sites and motifs. Genomes were categorized into *cooS*-negative and *cooS*-positive groups. Non-human gut genomes with putative WLP genes were also analysed. Metatranscriptomic analysis, genomic context analysis, metabolic function prediction and taxonomic and functional profiling were performed using tools including GTDB-tk, METABOLIC, eggNOG-mapper and CoverM to gain insight into their distribution and the metabolic traits of the pCODH-encoding genomes in the human gut microbiome.

The workflow of this study is depicted in Fig. 1. The publicly available genome collection, HumGut [1], was used as a source of human gut prokaryotic genomes (downloaded from https://arken.nmbu.no/~larssn/humgut/). In the database, >381 000 genomes derived from 3534 healthy human gut metagenomic samples were clustered into 30 691 representative genomes at 97.5 % sequence identity [1]. The previously reported bacterial genomes bearing genes for putative Ni-CODH (pCODH) and ACS without host association [2] were utilized and listed in Table S1, available in the online version of this article. Metatranscriptomic datasets derived from 110 different healthy individuals were downloaded from the NCBI SRA under the BioProject accession numbers PRJNA354235 and PRJNA707065 (Table S2).

### Detection of *cooS* in the human gut prokaryotic genomes

Ni-CODHs were searched from 30 691 representative human gut prokaryotic genomes, using *cooS* as genetic markers for Ni-CODH, respectively. The homology search was performed using NCBI blast+ 2.130.0 with cutoffs of an *E*-value of 10^−10^, a sequence length of 200 aa and a sequence identity of 30 %, using the following aa sequences as queries: clades A–H CODHs and mini-CODH from *Methanosarcina barkeri* CODH/ACS α subunit (WP_011305243.1), *Butyrivibrio* sp. CooS (WP_026514536.1), *Clostridium novyi* CooS (WP_039226206.1), *Carboxydothermus hydrogenoformans* CooSV (WP_011342982.1), *Thermococcus onnurineus* CooS (WP_012571978.1), *C. hydrogenoformans* CooSII (WP_011343033.1), *Deltaproteobacteria bacterium* RBG_16_49_23 CooS (OGP75751.1), Candidatus Atribacteria bacterium HGW-Atribacteria-1 CooS (PKP59679.1) and *Thermosinus carboxydivorans* mini CooS (WP_007288589.1), respectively [20,39]. The Ni-CODH clades of the detected CooS were determined by their aa sequence identity with representative CooS sequences. The detected putative CooS sequences were aligned using MAFFT v7.487 with the E-INS-i application [44]. To examine the motifs of CooS, previously used criteria [20] were adopted: no aa substitutions in two C-clusters comprising Ni, Fe and S; a B-cluster comprising cubane-type 4Fe-4S; and a d-cluster comprising an additional 4Fe-4S at the subunit interface [20,45, 46]. The information (e.g. IDs, CODH clades, length and identity) for the detected putative CODH (pCODH) is listed in Table S3. All genomes containing pCODHs are listed in Table S4.

A similar analysis was performed for CoxL as the marker for Mo-CODH with slight modifications. A homology search was performed using *Oligotropha carboxidovorans* CoxL (WP_013913730.1) as the query against the same HumGut dataset. The sequences that conserved the active site motif (AYRCSFR) for CO oxidation of Mo-CODHs [47] were regarded as the putative Mo-CODH.

### Phylogenetic tree construction

The taxonomic assignment of 30 691 prokaryotic genomes was performed using GTDB-tk v2.1.1, with the reference data version R207 [48]. The names of the phyla were corrected according to the method described [49]. Genome distances among the *cooS*-containing genomes were calculated using Mashtree v1.2.0 with 1000 replicates and a minimum depth value of 0 [50,51]. The obtained tree was then visualized using the R package, ggtree v3.2.1 [52]. Of the 3534 human gut metagenomic samples [1], the number of metagenomic samples, from which the bacterial genomes were detected with 95 % or higher identity, was displayed as a heatmap on the outer layer of the phylogenetic tree.

### Genome-based prediction of metabolic functions

To characterize the metabolic functions of the potential CO utilizer in the human gut, three groups of genomes were analysed using METABOLIC v4.0 [53]. The first group comprised the pCODH-encoding genomes detected in the HumGut database. The second group comprised the non-pCODH-encoding genomes of the human gut prokaryotes belonging to a family or order with pCODH-encoding genomes, which were detected in the HumGut database. The third group comprised the pCODH/ACS-encoding bacteria found in environments other than the gut. The proportions of genomes encoding functions in each genus were calculated for each group. From the HumGut database, we selected pCODH-lacking bacterial genomes belonging to orders or families containing pCODH-encoding bacteria, resulting in 194 genomes (the second group). We also utilized pCODH-encoding bacterial metagenome-assembled genome (MAG) datasets constructed by Inoue et al. [2]. Among the genomes, only the 554 genomes with pCODH/ACS genes but without ‘host-associated’ tags were used in this analysis. The accession IDs of the genomes are listed in Table S1.

### Characterization of gene compositions in the *cooS* genomic contexts

Protein-coding genes in the genomes were predicted using Prodigal v2.6.3, with default settings [54]. The gene functions were then predicted to the protein-coding sequences using eggNOG-mapper v2.1.5 [55]. For double-checking, a homology search of the protein-coding sequences against the clusters of orthologous gene (COG) database was performed using DIAMOND BLASTp v2.0.12 [56]. The KEGG orthology (KO) and COGs of 15 genes located in the upstream and downstream of the CooS loci were then extracted and are listed in Tables S5 and 6, respectively. Previously reported Ni-CODH-related COGs were also referred: AcsB (COG1614), CooF (COG0437 or COG1142), FNOR (COG1251), Ech (COG3260 and COG3261) and ABC transporter (COG0600, COG1116 and COG0715) [20]. The gene maps around *cooS* were visualized using the R package gggenomes v0.9.5.9000 [57].

### Genomic-based predictions of the degenerated WLP

Genomes that encode both pCODH and AcsB (K14138) were termed pCODH-/ACS-encoding genomes. All the KOs assigned to bacterial genomes in the human gut and other environments were listed, and the presence of each KO was checked in each genome to calculate the proportion of genomes carrying a given gene in each genome group. When two or more KOs were assigned to a single gene, all assigned KOs were considered for calculating the proportion. Since K00656 (PflD) contains members of pyruvate formate-lyase (PFL)-like proteins without PFL activity, proteins assigned as K00656 were aligned using MAFFT v7.487 with the E-INS-i application, and only the sequences that conserved the Cys-Cys active sites [58] were regarded as potential PFL-coding sequences. The proportions of the genomes carrying the given genes are listed in Table S7. In particular, the proportion of genomes carrying the following genes was extracted from the obtained results: CO metabolism-related genes (*cooC*, K07321; *cooF*, K00196), ACS subunits (*acsB–E*, K14138, K00197, K00194 and K15023; *cdhC*, K00193), WLP (*fhs*, K01938; *folD*, K01491; *metF*, K00297; and *metV*, K25007), Rnf subunits (*rnfA–G*, KO03612–17; *rnfC2*, K25008), *ech* (K15830, K15832), Hdr (*hdrA–C*, K03388–90), Mvh (*mvhD*, K14127) and PFOR (*por*, K03737; *porA–D*, K00169–72). In addition, formate-related genes were searched in the KO database and KEGG reaction database by inserting the keyword, ‘formate’. Hydrogenases were omitted from the results, and other genes were checked as the formate-related proteins: formate transporters (FocB, K03459; FocA, K06212; OxlT, K08177; YfdC, K21990; and FdhC, K21993), Fdh catalytic subunits (FdhA, K05299; FdhF, K22015; and FdoG, K00123) and the other subunits of Fdh (K00122–27, K22515 and K22516), PFL (K04069 and K00656) and other 55 genes. Genes that were not present in any genome were removed from the figure.

### Taxonomic and functional profiling of metatranscriptomes

Publicly available metatranscriptome datasets of healthy human were retrieved from the SRA database. Among the 110 datasets, 96 datasets were sourced from BioProject PRJNA354235, which includes samples from healthy adult men from the USA aged 40–75 years old [59], while 14 datasets were sourced from BioProject PRJNA707065, which includes samples from mothers and their 6-month-old infants from New Zealand or the United Kingdom under the NiPPeR study (https://www.nipperstudy.com/). Sequence reads were processed using KneadData v0.12.0 (http://huttenhower.sph.harvard.edu/kneaddata). In KneadData, the quality control pipeline was set as follows: FastQC v0.12.1 (https://www.bioinformatics.babraham.ac.uk/projects/fastqc/) was employed before and after quality controls, adaptor trimming was performed with default settings, reads were filtered by Trimmomatic v0.35 [60] with a sliding window size of 4 and a minimum quality score of 20, Tandem Repeats Finder v4.09 [61] was employed with default parameters and human host RNA (Hg38) and rRNA sequences (Silva v128) were removed using Bowtie2 v2.5.2 [62]. To evaluate the transcription of (i) pCODH genes, (ii) putative genes for the WLP and (iii) putative genes for Fdh, trimmed non-human reads from each dataset were subsequently subjected to the mapping onto (i) 1380 of human gut pCODH genes identified in this study; (ii) the 6 pCODH/ACS-encoding genomes with completeness >94 %, which are of * B. wexlerae* (HumGut_465), *Choladocola* sp003481535 (HumGut_2790), *Fusicatenibacter saccharivorans* (HumGut_64), UMGS1375 sp900066615 (HumGut_2058), *Faecousia* sp900546075 (HumGut_11723) and CAG-170 sp003516765 (HumGut_10793); and (iii) Fdh genes of *B. schinkii* (JANSWJ010000003.1 : 269667–271859) and *B. hydrogenotrophica* (NZ_CYXL01000001.1:c262854-260023), which are known to encode active Fdh [16,63], using CoverM v0.6.1 (https://github.com/wwood/CoverM). For the comparison of transcript levels among the pCODH in each dataset, the reads per kilobase of transcript per million reads mapped (RPKM) of each gene was calculated using CoverM with default settings. For the comparison of the transcription of coding genes within and between the datasets, transcripts per million values were calculated from the RPKM of each coding gene.

## Results

### The pCODH gene was possessed by 4.2 % of the human gut prokaryotic genomes

**Fig. 2.**
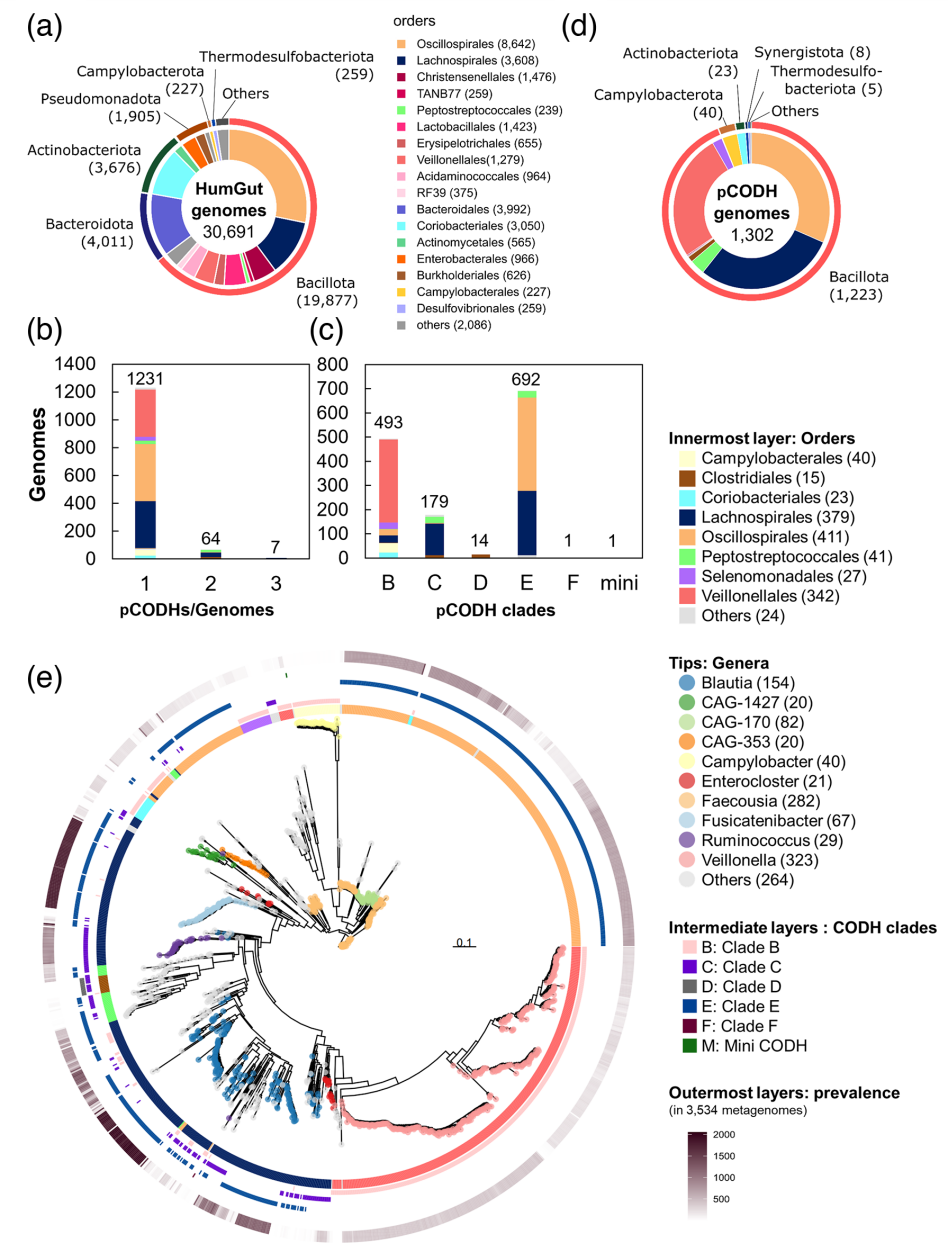
The putative Ni-CODH-encoding prokaryotic genomes in the human gut microbiome. (**a**) Taxonomic classification of publicly available 30 691 human gut prokaryotic genomes. The genomes were the representatives of more than 0.38 million genomes, clustered with 97.5 % nucleotide sequence identity [1]. The numbers in parentheses show those of genomes. (**b**) The number of genomes that possess one to three pCODHs. No genome possessed four or more pCODHs in the used dataset. (**c**) The number of genomes that possess a pCODH belonging to any of the six clades. (**d**) Taxonomic classification of the pCODH-encoding genomes. (**e**) Phylogenetic tree of the pCODH-encoding genomes. The tip colour represents the genus-level classifications. The heatmaps around the phylogenetic tree exhibit order-level taxonomic classifications; Ni-CODH clades B, C, D, E and F; and mini-CODH from the inner layer, respectively. The outermost layer shows the prevalence of each genome reported in the previous study [1]. The number indicates the metagenomic samples where each genome was analysed in 3534 metagenome datasets.

In the present study, we analysed the human gut prokaryotic genomes from the HumGut database [1]. The approximately 381 000 genomes derived from 3534 healthy human gut metagenome datasets were clustered into 30 691 genomes with 97.5 % sequence identity in the HumGut database [1]. Prior to exploring potential CO utilizer genomes in the human gut, 30 691 representative genomes were taxonomically classified using GTDB-tk [48], and 98 % of the gut microbial genomes belonged to the phyla Bacillota (19 877 genomes), Bacteroidota (4011), Actinobacteriota (3676), Pseudomonadota (1905), Campylobacterota (227) and Thermodesulfobacteriota (259) (Fig. 2a). The dominant orders in the Bacillota phylum were Oscillospirales (8642), Lachnospirales (3608), Christensenellales (1476), Lactobacillales (1423) and Veillonellales (1279) (Fig. 2a).

To identify potential CO utilizer genomes in the human gut, genes for the catalytic subunit of Ni-CODH clades A–G (*cdhA* and *cooS*) and mini-CODH were used as markers. As a result of homology search, 2505 aa sequences encoded by 2202 genomes were detected as candidate Ni-CODH homologues. Of these, the candidate Ni-CODH homologues were further filtered using the following criterion: no aa substitutions or deletions in the conserved motifs of the Ni-CODH of the five metal clusters and two acid–base catalysts [20]. Consequently, aa sequences encoded in 1380 genes of 1302 genomes were confirmed to conserve Ni-CODH motifs. Most of the genomes contained 1 pCODH gene (1231/1302 pCODH-encoding genomes), whereas the other genomes contained 2 or more genes (Fig. 2b). The detected 1380 pCODH genes belonged to clades B (493), C (179), D [14], E (692) and F [1] and mini-CODH [1] (Fig. 2c). Although putative Mo-CODH-encoding genomes were also surveyed using CoxL as a marker, no genome was found to possess putative gene for Mo-CODH in the human gut microbiome dataset.

Among the 1302 pCODH-encoding genomes, the phylum Bacillota comprised 1223 genomes (94 %). The remaining 79 genomes were identified as Actinobacteriota, Bacteroidota, Campylobacterota, Thermodesulfobacteriota, Pseudomonadota, Synergistota and Verrucomicrobiota (Fig. 2d, e). The 1223 Bacillota- and 79 other phyla-derived genomes belonged to 248 species, 82 genera and 8 phyla. Among Bacillota-derived genomes, 379, 341 and 411 belonged to Lachnospirales, Veillonellales and Oscillospirales, respectively; thus, a large proportion of the pCODH-encoding genomes were occupied by these three orders (Fig. 2d). The pCODH-encoding genomes accounted for 10.5, 26.7 and 4.8 % of the total human gut microbial genomes derived from the orders Lachnospirales, Veillonelalles and Oscillospirales, respectively. Within the order Lachnospirales, 38 genera possessed the pCODH genes, including *Blautia* (154/291; 154 pCODH-encoding genomes within 291 of the total genomes of the genus in the human gut microbiome), *Fusicatenibacter* (67/215), *Ruminococcus* (29/1194), *Enterocloster* (21/56), *Anaerobutyricum* (12/30) and UMGS1375 (10/11). Of these, *Blautia* was previously identified as possessing functional Ni-CODH and WLP [16] and was the only taxon to possess three pCODH genes (HumGut IDs: 13241, 3275, 5110, 12728, 20698, 11367 and 8982) (Fig. 2b). In *Blautia*, 24 of 154 pCODH-encoding genomes contained 2 or more pCODH genes. The Veillonelalles with pCODH genes consisted of 3 genera: *Veillonella* (323/443), *Megasphaera* (12/96) and F0422 (7/12). *Veillonella* is a ubiquitous bacterium found in the human body, such as in the oral cavity and gut [64]. The order Oscillospirales contains 13 genera that encode pCODHs in their genomes, including *Faecousia* (282/446), CAG-170 (82/179) and CAG-353 (20/60) (Table S4). Although Oscillospirales occupies a higher proportion of the human gut pCODH-encoding genome, most of these bacteria are uncultured; therefore, their physiological characteristics are largely unknown. pCODH-encoding genomes were also identified in the Bacillota orders Peptostreptococcales (41/239) and Selenomonadales (27/135) as well as in the Actinobacteriota order Coriobacteriales (23/3050) (Table S4).

To assess the distribution of potential CO utilizers in humans, we checked for the presence of pCODH-encoding genomes among the available 3534 datasets of healthy human gut metagenomes [1] (Fig. 2e). The pCODH-encoding genomes derived from the Lachnospirales genera *Fusicatenibacter* and *Blautia* were present in 48 and 26 % of the metagenome datasets, respectively. The Veillonelalles genus *Veillonella* was detected in 8.3 % of the metagenome datasets (Fig. 2e, Table S4). The Oscillospirales genera *Faecousia* and CAG-170 were detected in 13 and 18 % of the metagenome datasets, respectively. The above pCODH-encoding genomes appeared more prevalent than or as prevalent as the two prominent pCODH-lacking Bacteroidales genera, *Phocaeicola* and *Bacteroides* [1], which were detected in the 18 and 31 % metagenome datasets on average, respectively. In contrast, some pCODH-encoding genomes were present in only a limited number of the human gut metagenome datasets (Fig. 2e, Table S4). For instance, the pCODH-encoding genomes from the genus *Eubacterium* in the order Eubacteriales were found in only 0.2 % of metagenome datasets on average.

### Majority of pCODH-encoding genomes possessed putative genes for acetate production in the human gut

As most of the potential CO utilizers detected in this study have been uncultured and thereby uncharacterized, we predicted the genome-based metabolic functions of the potential CO utilizers bearing pCODH genes in the human gut microbiome using METABOLIC v4.0 [53]. Among the 23 metabolic functions predicted, the putative acetate production function was conserved among them (Fig. S1). In particular, the analysis revealed that 1162/1302 pCODH-encoding human gut microbial genomes, which correspond to 73/82 genera, contained putative genes for acetate production (*acdA*, *ack* and *pta*) for fermentation (Fig. S1). Exceptions were the Selenomonadales genera (*Centipeda*, *Mitsuokella* and *Selenomonas*), all of whose 27 genomes lacked the abovementioned genes for acetate production.

To evaluate whether these functional predictions were specifically applicable to human gut microbial genomes bearing pCODH genes, we compared the predicted metabolic functions of 1302 pCODH-encoding human gut microbial genomes to those of 194 pCODH-lacking human gut genomes that were closely related to the pCODH-encoding ones. All the predicted functions other than CO metabolism were observed in both types of genomes; thus, we could not predict any specific metabolic functions in the potential CO utilizers (Fig. S1). This suggests that CO utilization may be engaged in accessory functions that support the existing functions conserved in both pCODH-encoding and lacking human gut microbiomes.

### Eight *cooS* genomic contexts were identified in the human gut prokaryotic genomes

**Fig. 3.**
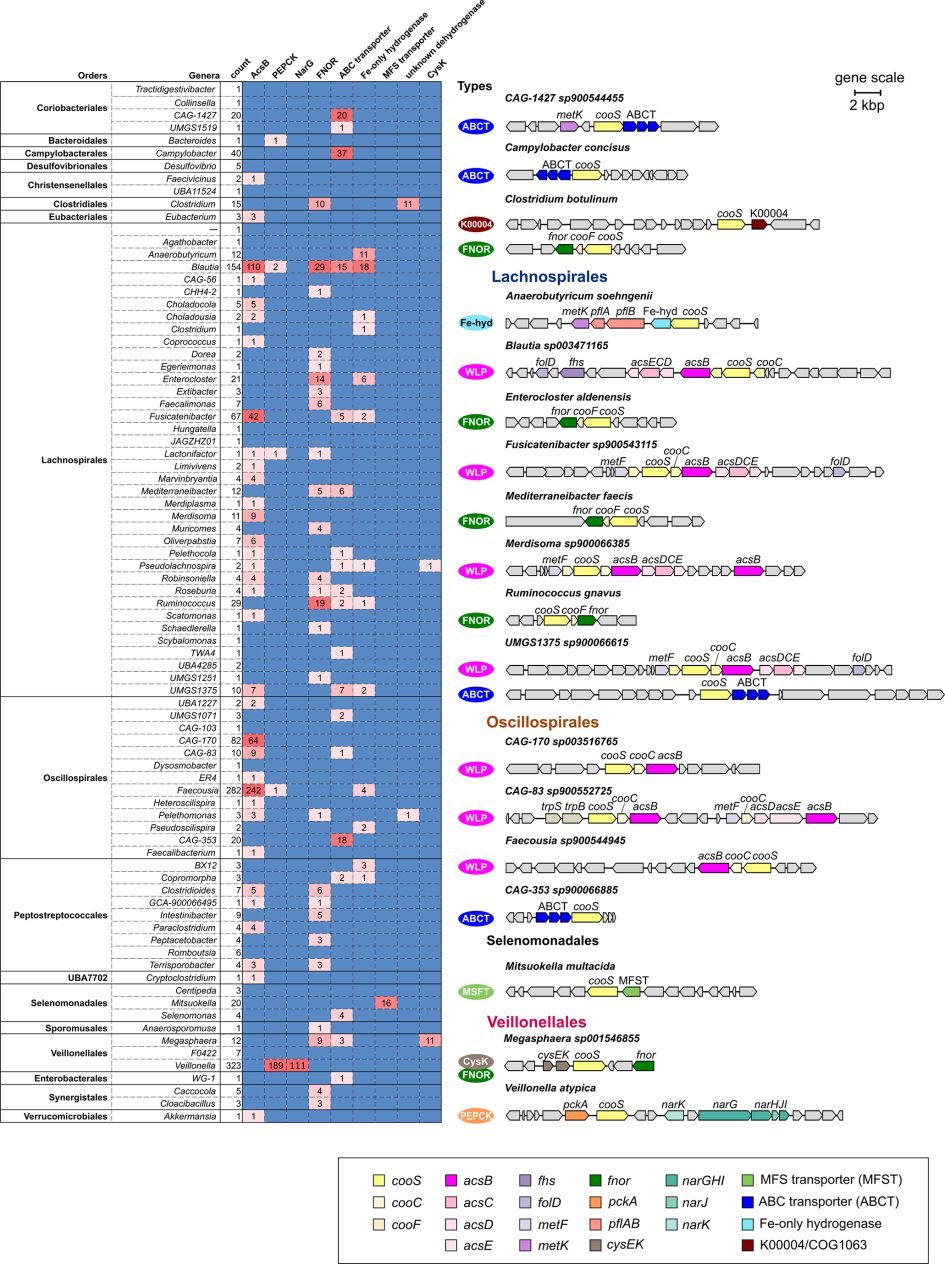
The genomic context of the putative Ni-CODH (*cooS*) in the human gut microbiome. The heatmap on the left shows the number of genes detected within the 15 genes upstream and downstream of *cooS* in the genomes of each genus. Genera with more than nine pCODH-encoding genomes were selected (red font), and the gene map around *cooS* of the genera is shown on the right. Each box represents protein-coding genes and their coded strands. The grey box represents the others and the hypothetical protein. *cooS*, carbon monoxide dehydrogenase catalytic subunit gene; *cooC*, carbon monoxide dehydrogenase maturation gene; *cooF*, ferredoxin-like protein-coding gene; *acs*, acetyl-CoA synthase gene; *fhs*, formyltetrahydrofolate synthase gene; *folD*, methylene-tetrahydrofolate dehydrogenase/cyclohydrolase gene; *metF*, methylene tetrahydrofolate reductase gene; *metK*, S-adenosylmethionine synthetase gene; *fnor*, flavin adenine dinucleotide-dependent NAD(P) oxidoreductase gene; *pckA*, phosphoenolpyruvate carboxykinase gene; *pfl*, pyruvate formate-lyase gene; *cys*, cysteine synthase gene; *nar*, nitrate reductase gene; MFS transporter, major facilitator superfamily transporter; ABC transporter, ATP-binding cassette transporters.

Genes that encode proteins involved in CO metabolism are often located close to *cooS* in the genome, forming genomic contexts that allow for the prediction of physiological roles of Ni-CODHs and CO metabolisms based on these genomic arrangements [20,39, 40]. To gain insight into the physiological roles of CO metabolism in the human gut microbiome, we analysed 15 genes located in the upstream and downstream regions of *cooS* in the 1302 human gut microbial genomes (Fig. 3, Tables S5 and S6). In total, 1150 KOs and 1285 COGs were assigned for genes within the 1380 *cooS* contexts in the 1302 genomes (Tables S5 and S6). Based on the predicted functions for KOs and COGs, genomic contexts were manually classified into eight types: WLP, phosphoenolpyruvate carboxykinase (PEPCK), FNOR, ABC transporter, Fe-only hydrogenase, major facilitator superfamily (MFS) transporter, uncharacterized dehydrogenase and cysteine synthase. Genes for previously reported CO-oxidizing coupled respiratory reactions, such as Ech genes, were not found in the human gut pCODH-encoding genomes. Below, we describe the predicted physiological functions and detailed phylogenetic distributions of the genomic context types (Fig. 3).

The WLP type, characterized by the presence of the gene for ACS β subunit (AcsB; K14138, COG1614), was the most prevalent in the human gut microbial genomes, being detected from 593 of the 1380 *cooS* contexts. The WLP context was observed in Oscillospirales (8/13 genera, that is, 8 pCODH-encoding genera within the total genera of the order; 323/411 genomes, that is, 323 pCODH-encoding genomes within the total genomes of the order), Lachnospirales (18/38 genera, 198/379 genomes), Peptostreptococcales (4/9 genera, 13/41 genomes) and Eubacteriales *Eubacterium* (3/3 genomes). All genera conserved the consecutive gene arrangement of *cooS-cooC-acsB*, except for the Peptostreptococcales genera, which had the gene arrangement of *cooS-cooC-fhs* and *acsB* at 10 genes downstream of *cooS* (Tables S5 and S6). *acsCDE* was found in the *cooS* contexts of the Lachnospirales and Peptostreptococcus genera but not in seven out of eight Oscillospirales genera (Fig. 3, Tables S5 and S6).

The second type is the PEPCK, which has been unpreceded in *cooS* genomic contexts. PEPCK (*pckA*; K01610, COG1866) is a phosphoenolpyruvate (PEP) carboxykinase that catalyses the conversion of PEP, CO_2_ and ADP to oxaloacetate and ATP and vice versa. The PEPCK type was found among the genomes of Veillonelales genus *Veillonella* (189/342 genomes). Gene composition varied among PEPCK-type genomic contexts. Of the 189 PEPCK-type genomic contexts of *Veillonella*, 144 included a putative *oxyR*, which encodes a transcriptional regulator of the LysR family (K04761, COG0583) and responds to oxidative stress [65] in the upstream region adjacent to *cooS*. Genes for nitrate reduction (*narGHIJK*) were also observed in 111 *Veillonella* PEPCK-type genomic contexts, of which 78 genomes also included *oxyR* (Fig. 3).

The third is FNOR type, which has been predicted to be associated with CO utilization in the previous studies [2,20, 66]. FNOR (COG1251) is known to be often present in CO utilizers and is involved in energy conservation through CO oxidation associated with the reduction of NAD(P)^+^ [66]. In the human gut microbial genomes, putative FNOR genes were found in 138 *cooS* contexts of bacteria, including Lachnospirales (15/38 genera, 92/379 genomes) and Peptostreptococcales (5/9 genera, 18/41 genomes) (Fig. 3).

The following types have been reported previously; however, their physiological functions in CO metabolism are unknown: putative ABC transporters (K02049–51, COG0715-COG0600-COG1116) were found in the genomic context of various human gut bacterial genomes, such as Lachnospirales (9/38 genera, 40/379 genomes), Campylobacterales *Campylobacter* (37/40 genomes) and Selenomonadales *Selenomonas* (4/4 genomes) (Fig. 3). The putative Fe-only hydrogenase large subunit (COG4624) was encoded in the genomic context of the genomes in Lachnospirales (9/38 genera, 43/379 genomes), Oscillospirales (2/13 genera, 6/411 genomes) and Peptostreptococcus (2/9 genera, 4/56 genomes) (Fig. 3, Table S6). The COG4624 gene was located downstream region adjacent to *cooS* especially in 24 of the 57 *cooS* genomic contexts with COG4624, including the contexts from Lachnospirales *Anaerobutyricum*. COG4624 in this organism was predicted as group A2 FeFe hydrogenase in METABOLIC. In 17 of these, *pflAB* (K04069 and K00656) was found within the genomic context of *cooS* (Fig. 3). In addition to the putative Fe-hydrogenase-type context, genes assigned as COG4624 were also found in the WLP-type context in 27 genomes.

‘MFS transporter’, ‘functionally uncharacterized dehydrogenase’ and ‘cysteine synthase’ were relatively minor types, as they occupied fewer than 2 % of the pCODH-encoding genomes and were observed in specific taxa. The MFS transporter is responsible for importing or exporting a wide range of substrates across the membrane using a substrate concentration gradient. The putative MFS transporters encoded in this context were predicted as COG2223 (NarK, nitrate transporter) and K08177 (OxlT, oxalate/formate antiporter) in Selenomonadales *Mitsuokella* (16/20 genomes) (Fig. 3). In the functionally uncharacterized dehydrogenase context type, putative genes for K00004 (butanediol dehydrogenase) and COG1063 (threonine dehydrogenase or related Zn-dependent dehydrogenase) were found in the downstream region adjacent to *cooS* in Clostridiales *Clostridium* (11/15 pCODH-encoding genomes), whereas putative genes for hydrogenase nickel incorporation protein (*hypAB*, K04651 and K04652), which may be involved in maturation of Ni-CODH, were present upstream of *cooS*. The putative cysteine synthase (CysK; K01738, COG0031) gene was mainly found in Veillonellales *Megasphaera* (11/12 genomes).

### The Fdh-lacking WLP is predicted to be common in the human gut microbiome

**Fig. 4.**
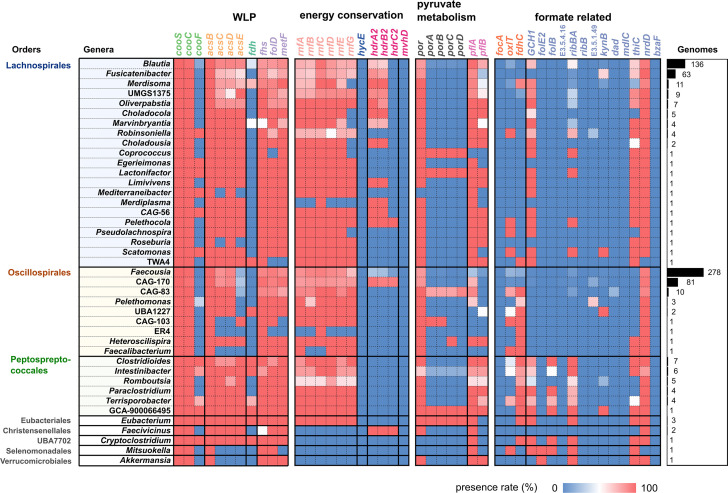
The presence rates of the putative WLP genes and other related genes in the human gut genomes. Genomes possessing *cooS* and *acsB* were analysed to reveal the presence of putative WLP-related and other genes. The presence rates are displayed for each genus on a heatmap. *cooS*, carbon monoxide dehydrogenase catalytic subunit gene; *cooC*, carbon monoxide dehydrogenase maturation gene; *cooF*, ferredoxin-like protein-coding gene; *acsBCDE*, acetyl-CoA synthase gene; *fdh*, formate dehydrogenase catalytic subunit gene; *fhs*, formyltetrahydrofolate synthase gene; *folD*, methylene-tetrahydrofolate dehydrogenase/cyclohydrolase gene; *metF*, methylene tetrahydrofolate reductase gene; *rnf* ferredoxin:NAD^+^ oxidoreductase gene; *hycE*, energy-converting hydrogenase catalytic subunit gene; *hdr-mvh*, methylenetetrahydrofolate reductase gene; *por*/*porABCD*, pyruvate: ferredoxin oxidoreductase *pfl*, pyruvate formate-lyase gene. *focA*/*oxlT*/*fdhC*, genes are involved in formate transport. Other genes, genes involved in formate consumption and metabolism. The right bar represents the number of genomes in each genus.

As the WLP-type genomic context was the most prevalent among the 1302 pCODH-encoding genomes in the human gut, we further investigated whether the human gut prokaryotes bearing the WLP context indeed possess the entire gene set of acetyl-CoA synthesis through the WLP. Since WLP is known to be functional even in prokaryotes of which *cooS* and *acsB* are not located in close proximity within the genomes [35], we here referred to the 667 genomes bearing both *cooS* and *acsB* as pCODH/ACS-encoding genomes regardless of whether the genomes retain the *cooS* and *acsB* genes as the WLP type context. The presence of other WLP genes such as *cooCF*, *fdh*, *fhs*, *folD*, *metF* and *acsCDE* was surveyed in all the genomes containing genes for pCODH/ACS (667 genomes) (Fig. 4).

From more than 88 % of the pCODH/ACS-encoding genomes, almost the full set of putative WLP genes, including *acsCD*, *cooC*, *fhs*, *folD* and *metF*, was detected (Figs. 4 and S2). Interestingly, *acsE*, a bacterium-specific gene for the methyltransferase subunits of ACS, was not detected in 78 % of the Oscillospirales genomes (7/9 genera) (Fig. 4), whereas *acsE* was detected in the other pCODH/ACS-encoding genomes. Although a nearly full set of WLP genes was detected, we found that the putative Fdh genes for formate synthesis (*fdhA*, *fdhF* and *fdoG*) were not detected in 95, 96 and 79 % of the pCODH/ACS-encoding genomes, respectively (Fig. 4), and none of the putative Fdh genes were detected in 79 % of the genomes. It is noted that 52 out of 114 putative Fdh sequences (51 of them were from the order Oscillospirales) were also predicted as glutamate synthase (K00266). A previous study reported that two *Blautia* species are capable of CO metabolism in the presence of external formate even though the bacteria lack Fdh gene/activity [16]. In this study, the presence of Fdh genes was also checked in the 554 pCODH/ACS-encoding genomes found in other environments [2] (Fig. S2). It revealed that putative gene sets for the Fdh-lacking WLP are also present in the genomes of other environments. However, compared to the human gut microbial genomes pCODH/ACS-encoding genomes, putative Fdh genes (*fdhA*, *fdhF* and *fdoG*) were missing in a smaller proportion of bacteria of other environments (28, 84 and 17 %, respectively) (Fig. S2), and none of the putative Fdh genes was observed in 14 % of the genomes. Thus, the lack of genes for formate synthesis might be a feature highly enriched in the human gut potential CO utilizer genomes. Similarly, *cooF*, a putative gene for Fd-like protein, was not detected in 91 % of the pCODH/ACS-encoding genomes (accounting for 26/42 genera), whereas *cooF* was absent in 53 % of the bacterial genomes isolated from other environments (Figs. 4 and S2).

The above analyses suggest that there may be CO_2_-independent sources of formate for human gut pCODH/ACS-encoding bacteria if they utilize the WLP. To estimate how formate is obtained in that case, we surveyed the genes predicted to be involved in alternative formate synthesis or in import in the pCODH/ACS-encoding human gut microbial genomes (Fig. 4). The Lachnospirales (16/21 genera, 145/239 genomes), a few Oscillospirales genera (2/9 genera), all Peptostreptococcus genera (5/5 genera, 22/26 genomes) and Verrucomicrobiales genera Akkermansia (1 genome) contained putative *pflB* gene of which product is involved in formate synthesis from pyruvate (Fig. 4). Three Oscillospirales genera that lacked homologues of both *fdh* and *pfl* in their genomes possessed putative genes for formate transporters, *fdhC* (56/291 genomes) (Fig. 4). Two Lachnospirales (*Merdiplasma* and *Fusicatenibacter*) and two Oscillospirales genera (CAG-170 and *Faecousia*) were found not to possess homologues of these genes. Instead, from genomes of these genera, putative genes encoding proteins involved in formate-producing/utilizing reactions such as GTP degradation (GCH1, *ribBA*) and thiamine metabolism (*thiC*) were detected (Fig. 4).

To gain deeper insights into the functions specifically predicted from the human gut pCODH/ACS-encoding genomes, we compared all the genes in the genomes of the human gut bacteria and other environmental bacteria encoding pCODH/ACS (Fig. S1, Table S7). We observed that putative genes involved in carbohydrate and saccharide degradation were more prevalent in pCODH/ACS-encoding genomes of the human gut than in other environments (Fig. S1, Table S7). In addition to the lack of homologues of Fdh genes, enriched gene compositions putatively for carbohydrate and saccharide degradation might be a key feature in the human gut microbial pCODH/ACS-encoding genomes. Associated with the enriched genes putatively for carbohydrate and saccharide degradation that are related to glycolysis supplying pyruvate, we found an enrichment of putative genes for pyruvate metabolisms. PFOR (*por*) is known to be involved in WLP by supplying Fd_red_ from pyruvate oxidation [19]. Putative PFOR gene is present in 90 % of the human gut pCODH/ACS-encoding genomes, in addition to putative *pflB* in 53 % of those genomes. These genes were less frequently observed in pCODH/ACS-encoding genomes in other environments: putative *por* and *pflB* were present in 71 and 19 % of the genomes in other environments, respectively (Fig. S2).

Not only putative genes for carbohydrate/saccharide degradation (above), but also the genes for putative Rnf complex (*rnfABCDEG*) were detected in a high proportion of pCODH/ACS-encoding genomes in the human gut microbiome (>73 %). The putative FeFe group B hydrogenase was encoded by 64 % of pCODH/ACS-encoding genomes (Fig. S2). Although some FeFe hydrogenases are known to be an electron-bifurcating enzyme that provides electrons to Fdh of some CO-utilizing acetogens [30,67], the co-occurrence of putative genes for FeFe group B hydrogenase with putative Fdh genes was not statistically significantly large (Fig. S3).

### The pCODH transcripts were detected in 97.3 % of metatranscriptomic datasets

**Fig. 5.**
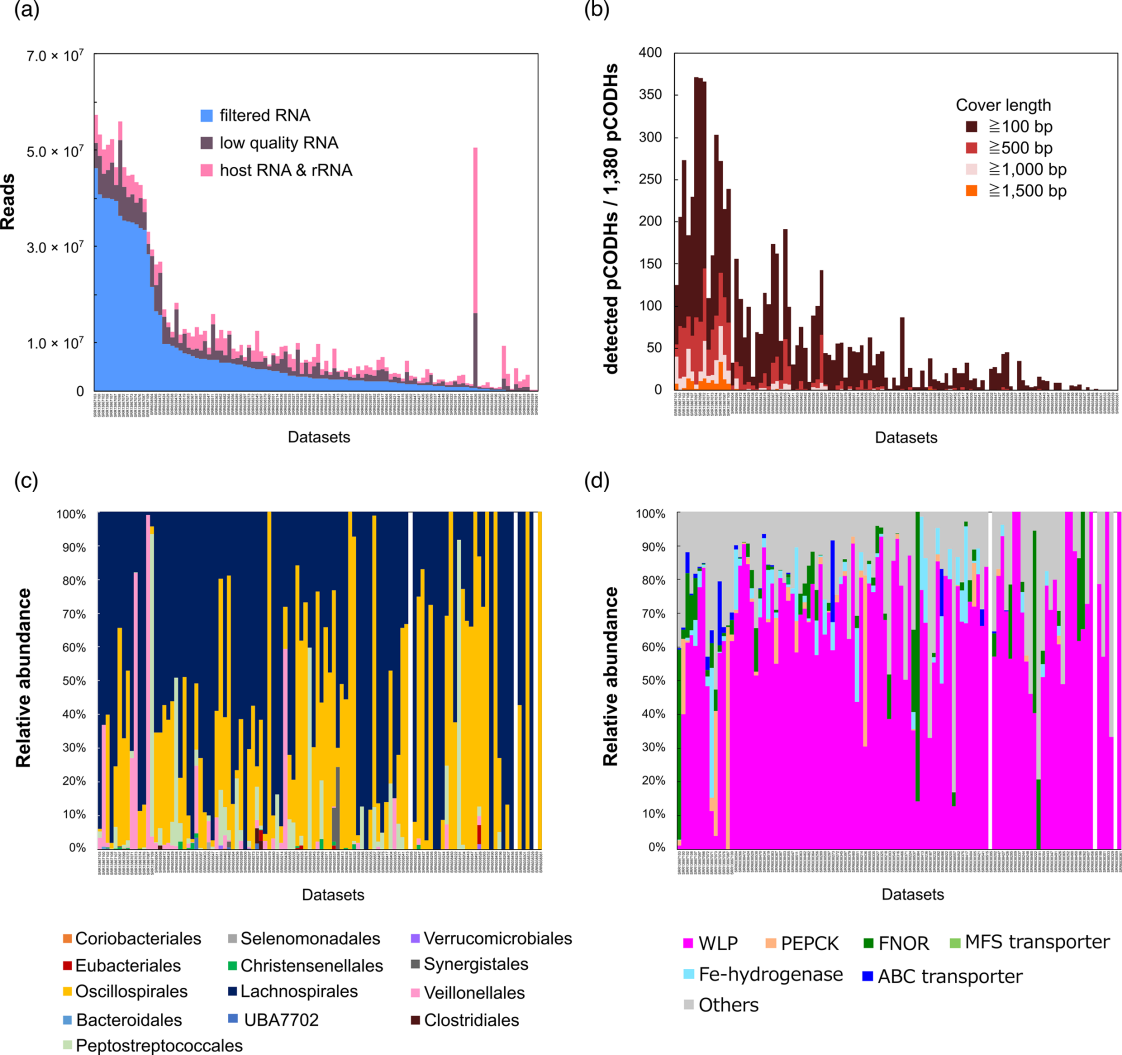
Distribution and diversity of putative Ni-CODH transcripts in the healthy human gut metatranscriptomic datasets. (**a**) Composition of sequence reads in the 110 publicly available metatranscriptomes in the healthy human faeces. (**b**) Number of detected pCODH genes among 1380 non-redundant pCODH sequences. (**c**) Relative abundances of host bacteria of the detected pCODH sequences in the metatranscriptomic datasets. (**d**) Relative abundances of pCODH types in the metatranscriptomic datasets. In the x-axis of the graphs, datasets with a greater number of filtered RNA are aligned from left to right.

To investigate whether the pCODH genes detected in this study are transcriptionally active in the human gut microbiome, metatrascriptomic datasets from 110 healthy human individuals [59] were analysed. Following quality control, 7.8±1.2 million reads were obtained per dataset on average (Fig. 5a). Filtered metatranscriptome reads were then subjected to mapping onto 1380 pCODH gene sequences of the human gut microbial genomes. It was found that pCODH genes were transcribed in 107 out of 110 metatranscriptome datasets, with an average of 1127 reads mapped to the pCODH gene sequences (Fig. 5b). The number of detected pCODH genes correlated with the total number of reads per dataset (*r^2^*=0.76). Read mappings with a cover length of >1500 nt for pCODH genes were observed only in the datasets containing more than 28 million filtered reads (Fig. 5b). The human gut prokaryotes, of which the pCODH genes were transcribed, were assigned to the 13 orders from 7 phyla (Fig. 5c). None of the reads from the pCODH-encoding genomes of the phyla Campylobacterota, Desulfobacterota and Proteobacteria were detected in the healthy human metatranscriptome datasets. At the order level, transcript reads of the pCODH genes were detected from Lachnospirales, Oscillospirales, Peptostreptococcales, Veillonellales, Synergistales and other 8 orders with 2.9×10^5^, 1.7×10^5^, 2.6×10^4^, 1.8×10^4^ and 1.8×10^3^ and 55 to 815 RPKMs of the pCODH transcript reads across the 110 datasets on average, respectively (Fig. 5c). At the genus level, pCODH transcript reads from total 66 genera were detected (Fig. S4). In Lachnospirales, the genera *Blautia*, *Choladocola*, *Fusicatenibacter* and UMGS1375 accounted for 37.9, 19, 12 and 7.9 % of pCODH transcript reads, respectively. In Oscillospirales, the genera *Faecousia* and CAG-170 accounted for 65 and 26 % of the pCODH transcript reads, respectively. In Peptostreptococcales, 58 % of pCODH transcript reads were from the genus *Intestinibacter*. In Veillonellales, 99.7 % of pCODH transcript reads were derived from the genus *Veillonella*. Regarding the genomic context-based functional prediction, pCODH transcript reads derived from six out of eight types (WLP, PEPCK, FNOR, ABC transporter, Fe-hydrogenase and MFS transporter) were detected in the human gut metatranscriptomes (Fig. 5d). The most abundant type was the WLP, accounting for 66 % of pCODH transcript reads on average, while the average relative abundances of other types ranged from 0.02 to 5.0 % (Fig. 5d).

**Fig. 6.**
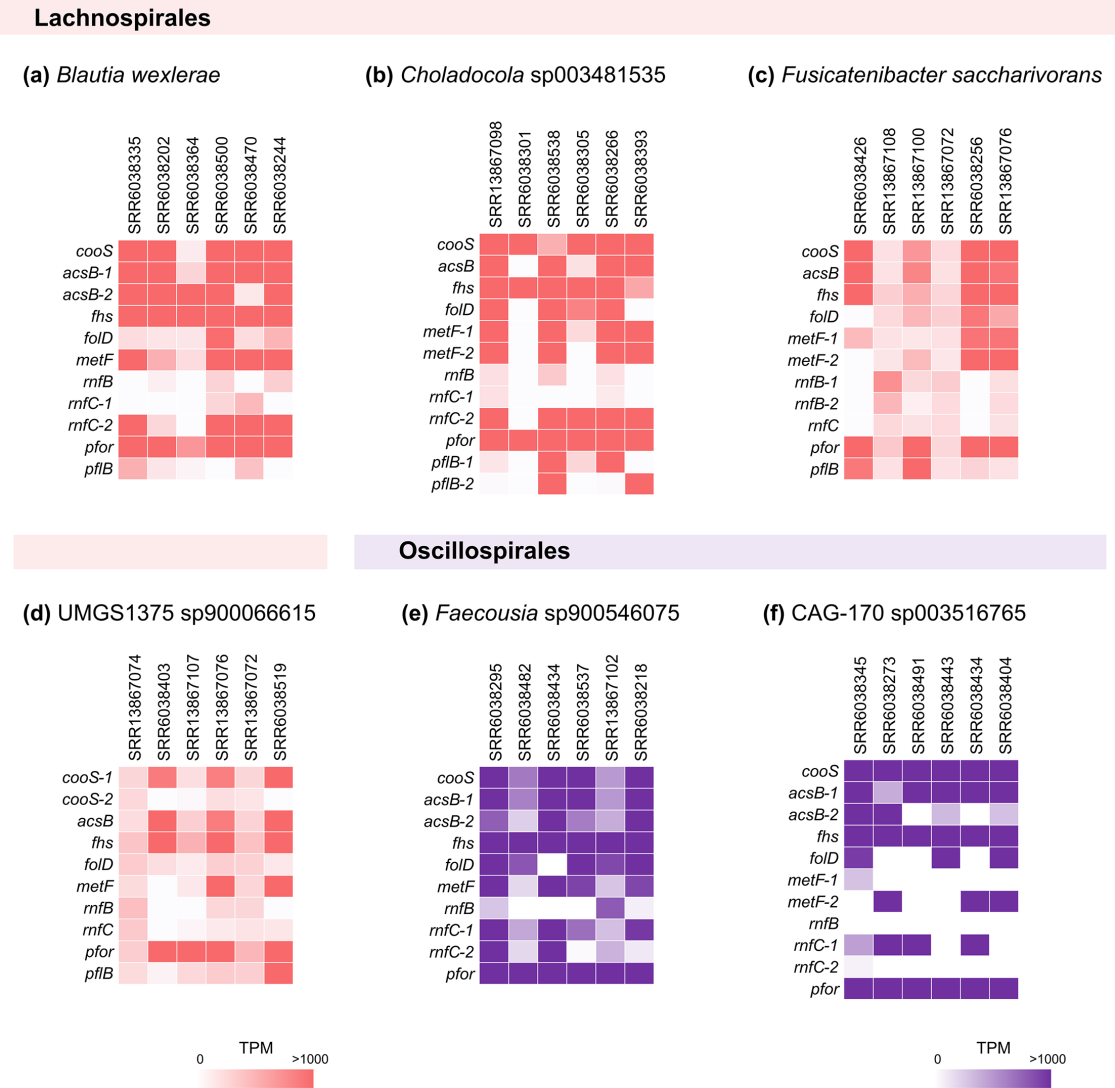
Abundance of the putative WLP-related transcripts in the human gut metatranscriptomes. The heatmaps illustrate the relative abundances of the putative WLP transcripts derived from 7 different pCODH-bearing genera that were most prevalently detected in the 110 human gut metatranscriptomes. Genomes with more than 94 % completeness were selected as references to map the 6 metatranscriptomic datasets containing the pCODH reads for the selected 6 genomes. The seven genomes were derived from (**a**) *Blautia wexlerae*, (**b**) *Choladocola* sp003481535, (**c**) *Fusicatenibacter saccharivorans*, (**d**) UMGS1375 sp900066615, (**e**) *Faecousia* sp900546075 and (**f**) CAG-170 sp003516765.

To determine if the entire set of putative genes for WLP is transcribed in the human gut microbiome, mapped transcript reads onto the putative WLP-related genes were investigated. Among the species with pCODH/ACS genes, those with the largest number of pCODH transcript reads and genomes with completeness greater than 94 % were used as the references for read mapping. As a result, the transcript reads of all the putative WLP genes (*cooS*, *acsB*, *fhs*, *folD* and *metF*) from genomes of six species (*B. wexlerae*, *Choladocola* sp003481535, *Fusicatenibacter saccharivorans*, UMGS1375 sp900066615, *Faecousia* sp900546075 and CAG-170 sp003516765) were detected in the human gut metatranscriptome, clearly indicating that the genes for WLP are transcribed in the human gut (Fig. 6). Metatranscriptome reads were also subjected to mapping onto *fdhF* from *B. schinkii* and *B. hydrogenotrophica*, resulting in the detection of transcript reads of *B. hydrogenotrophica fdhF* in 4 out of the 110 datasets, although the cover length was shorter than 500 nt in all the 4 datasets. Both transcripts for putative PFOR and PFL were also detected (Fig. 6a–d). In *B. wexlerae*, *Choladocola sp003481535*, *F. saccharivorans* and UMGS1375 *sp900066615*, the putative PFOR transcripts were detected at higher levels than putative PFL, with an average fold difference of 63, 7.6, 7.9 and 5.1, respectively (Fig. 6a–d).

## Discussion

Our analysis uncovered phylogenetically diverse pCODH-encoding genomes, belonging to 248 species and 82 genera across 8 phyla (Fig. 2). Furthermore, the transcripts of pCODH genes derived from 66 genera, 5 phyla, were identified in 107 of 110 metatranscriptome datasets derived from a healthy human with various nationalities and ages (ranging from 6 months to 75 years old). This suggests that the CO-utilizing pathways are transcriptionally active and, if active in enzymatic level, may constitute a CO-consuming function of the human gut environment. To date, only 2 human gut prokaryotes have been experimentally verified as CO utilizers [16], and pCODH was bioinformatically identified in 280 genomes from genus *Blautia* [16] and 43 genomes from gastrointestinal acetogenic strains from orders including Lachnospirales and Clostridiales [43]. Therefore, our findings extensively expand the catalogue of prokaryotes bearing pCODH genes in the human gut environment as our analysis revealed that genomes from orders such as Oscillospirales and Veillonellales, of which CO utilizers have not fully been unveiled, constitute large proportions of the pCODH-encoding genomes.

Our findings are not limited to phylogenetic diversity but also raise a hypothesis on potential functional diversity of CO utilization in the human gut microbiome. The previously identified CO-mediated metabolic pathway in the human gut-derived prokaryotes was the WLP [16]. However, the pCODH genomic contexts identified in the human gut microbiome can be divided into eight distinct types based on gene compositions around *cooS*. If pCODHs are expressed and play certain physiological roles with the enzymes encoded in the neighbouring genes, as previously described [2,20, 39, 40], the diverse prokaryotic taxa of the human gut microbiome are estimated to be involved in CO utilization through various pathways. However, none of the previously identified genes associated with CO-oxidizing energy conservation, typically discussed in the context of CO detoxification [29] and/or bacterial persistence [21,26], were found in the pCODH genomic contexts of the human gut microbes. Considering the low redox potential and low concentration of CO in the human gut, it is plausible that Ni-CODH is not used for CO detoxification, but rather for biosynthesis if pCODH is functional in these human gut microbes.

**Fig. 7.**
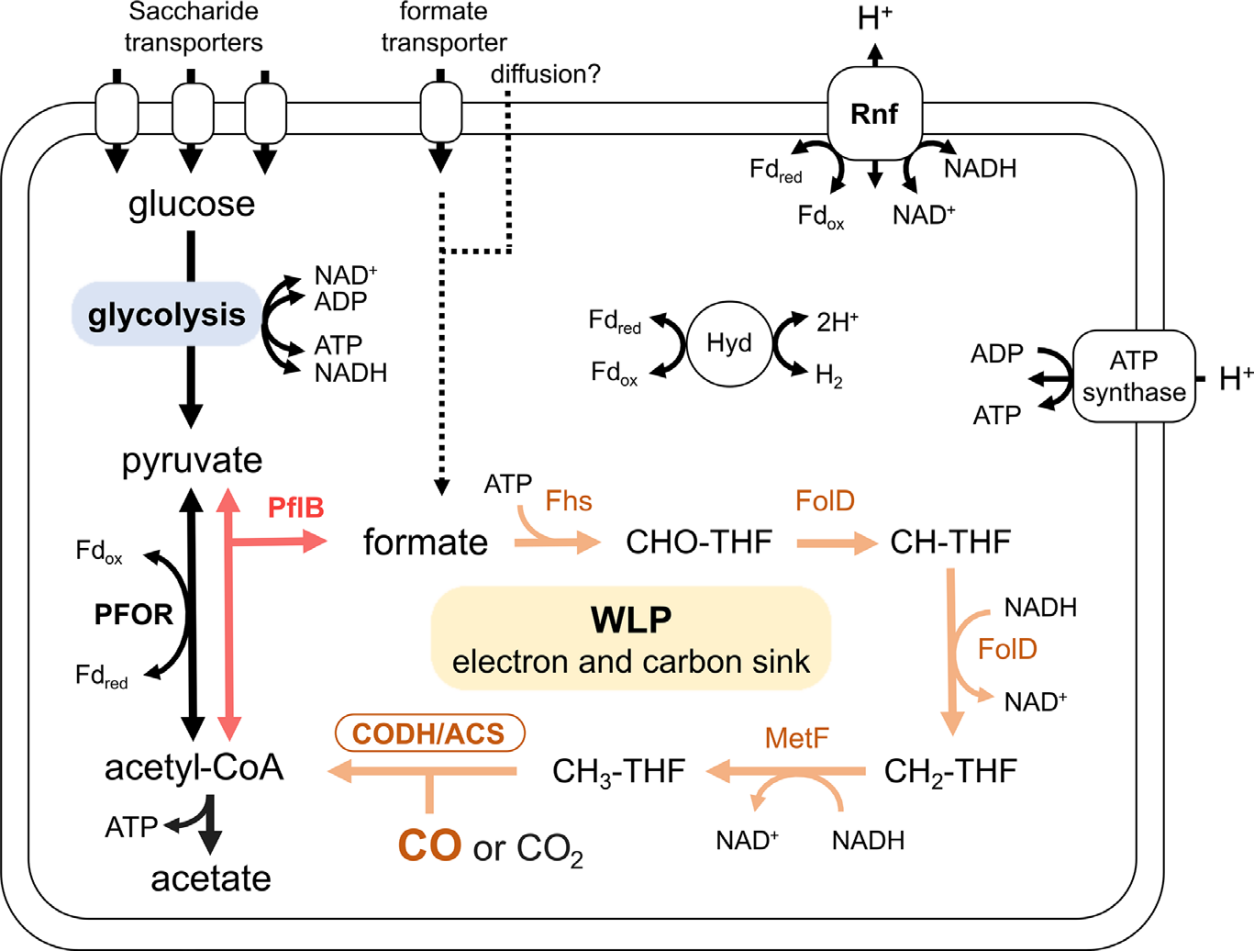
Genome-based metabolic reconstructions of the Fdh-lacking WLP in the human gut. A schematic of Fdh (formate dehydrogenase)-lacking WLP identified in the human gut bacteria, including genera *Blautia* from Bacillota order Lachnospirales. The figure shows an example of metabolic pathways. Pyruvate that is mainly obtained via glycolysis is converted to acetyl-CoA and formate by PFL (pyruvate formate-lyase) (red lines). Formate is then progressively converted to methyl moiety in the WLP (orange lines) using formyl-tetrahydrofolate synthase (Fhs), methylene-tetrahydrofolate dehydrogenase/cyclohydrolase (FolD) and methylene-tetrahydrofolate reductase (MetF). CO and CH_3_ are then incorporated to acetyl-CoA with CO dehydrogenase/acetyl-CoA synthase (CODH/ACS). The WLP may function as a sink for electrons and/or carbons, potentially supporting energy conservation and the uptake of CO.

We observed that putative genes for WLP were the most prevalent in the human gut microbial genomes and the metatranscriptome of human gut microbes, such as those of Oscillospirales, Lachnospirales and Peptostreptococcales. Almost all the putative WLP genes were identified in the pCODH/ACS-encoding genomes of the human gut microbiome. In particular, pCODH transcripts from *Blautia* species were detected from 78 % (86/110) of the metatranscriptome datasets of the human gut microbiome (Fig. S4), suggesting the wide contribution of this genus for CO consumption in the human gut. However, homologues of Fdh genes were not detected in a large number of the pCODH/ACS-encoding human gut microbial genomes (Fig. 4). Previous cultivation experiments have demonstrated that human gut CO utilizers, *B. luti* and *B. wexlerae*, in the order Lachnospirales lack both Fdh gene and its activity, but the resting cells of these bacteria possess functional WLP, which utilizes extracellular CO and formate [16]. Consistent with the previous publication [16], the genomes of *Blautia* derived from the human gut microbiome possess putative gene sets for Ni-CODH and WLP but lack homologues of Fdh gene. These *Blautia*-derived genomes including those of *B. luti* and *B. wexlerae* possess putative genes for PFL, which is known to synthesize formate and acetyl-CoA from pyruvate (Fig. 7). If putative PFL was indeed expressed and functional in this genus, glycolysis synthesizing pyruvate could be one of the sources for carbon for the WLP of *Blautia* species, including *B. luti* and *B. wexlerae* (Fig. 7). However, in *Blautia* strains, putative PFOR may also contribute to the production of acetyl-CoA from pyruvate. Therefore, the biological role of WLP may not be for acetyl-CoA production associated with CO_2_ fixation but rather for providing a pool of electrons and carbon in the acetate-rich environment of the human gut [68], potentially conserving energy in the methyl branch [38] and/or fixing CO (Fig. 7). This assumption should be tested experimentally, but our data raised a possibility that their WLP may have been modified to a ‘heterotrophic form’ that obtains carbon from organic compounds instead of CO_2_.

Previous genomic studies unveiled that several human gastrointestinal acetogens, including species from Lachnospirales (*B. producta* and *M. formatexigens*) and Clostridiales (*C. bovifaecis* and *C. difficile*), lack homologues of Fdh genes but retain putative gene sets for WLP [42,43, 69] although their CO-utilizing ability has not experimentally been confirmed. This study indicates that loss of putative Fdh genes in pCODH/ACS-encoding genomes is not limited to *Blautia*, *Marvinbryantia* and *Clostridium* but more prevalently distributed to diverse taxa of the human gut potential CO utilizers, represented by the 526 genomes from 32 genera. In 53 % of these pCODH/ACS-encoding genomes, the ‘heterotrophic WLP’ system using PFL, which could link glycolysis and the WLP, may be one option for carbon acquisition as described above for *Blautia* species. Importantly, many human gut genomes with genes for such ‘heterotrophic WLP’ retain not only putative genes for PFL but also putative genes for carbohydrate degradation to a greater extent than those from other environments (Fig. S1, Table S7). If products of these genes had the predicted activities, these enzymes could provide substrates for glycolysis, and thereby pyruvate for PFL-mediated formate synthesis, in the human gut potential CO utilizers. This alternative pathway for formate synthesis may be one of the reasons why homologues of Fdh genes have been lost from many of the genomes of acetogens in the gut environment [43]. In addition, the previous findings by Levine et al. [41], which observed higher CO consumption activity of faeces samples in the presence of glucose (0.7 ml/h, g faeces) than in the absence of glucose (0.2 ml/h, g faeces), might at least partially be caused by the potential heterotrophic WLP estimated in the human gut microbiome. Regarding the other routes for formate acquisition, 141 genomes possessed putative Fdh genes [70], while genomes lacking both putative Fdh and PFL were also present. If the intracellular synthesis of formate is indeed lacked in these bacteria, they may import formate from extracellular fractions as the substrate for the WLP by using the formate transporters or by cross-feeding [71].

CO utilization pathways other than the WLP may also be present in the human gut. A novel type of Ni-CODH gene context, encoding putative PEPCK involved in gluconeogenesis and putative nitrate reductase (NarKGHJI), was identified in 190 genomes of the genus *Veillonella* (Fig. 3). Their transcripts for pCODH were detected in 35.5 %(39/110) of the metatranscriptome datasets, indicating genes for pCODH of this genus were transcriptionally active in the human gut. In *Veillonella*, a biochemical link between CO oxidation and gluconeogenesis/nitrate reduction could be a possible explanation for CO utilization, while our analysis solely relies on the predictions based on the genomic context. In addition to *Veillonella*, pCODH transcripts from *Anaerobutyricum* species, of which genomic contexts were the Fe-only hydrogenase type, were detected in 38.2 %(42/110) of the metatranscriptome (Fig. S4). In this type, CO oxidation may be associated with production of hydrogen and reduction of electron carriers such as Fd_ox_ if pCODH and Fe-hydrogenase were expressed and active in the bacteria. Since the reconstructed pathways described above are conjectural based only on the metagenome-assembled genomes and the metatranscriptomes, further experimental verifications are necessary.

This study revealed the phylogenetic diversity of pCODH-encoding genomes and a variety of Ni-CODH genomic contexts in the human gut microbiome. Since trace amounts of CO are constantly produced in the human body [8], at least a part of the prokaryotic species estimated to have Ni-CODH in this study, such as *Blautia* spp. [16], may utilize CO for their viability in the human gut microbiota, thereby affecting CO concentration in the human gut. Importantly, our analyses did not detect genes for hydrogenogenic CO oxidation, suggesting that prokaryote-mediated CO consumption in the human gut is not for CO removal as the form of CO_2_ through energy conservation, known as an environmental safety valve [28]. Although which prokaryotes are indeed capable of consuming CO and how much CO is utilized by those prokaryotes in human gut should be confirmed experimentally in the future, shedding light on human gut CO utilizers would help elucidate the microbial ecosystems in gut environments in humans.

## supplementary material

10.1099/mgen.0.001285 Uncited Fig. S1.

10.1099/mgen.0.001285 Uncited Table S1.

## Abbreviations

ACS: acetyl-CoA synthase
CoA: coenzyme A
CODH: CO dehydrogenase
COG: clusters of orthologous gene
Ech: energy converting hydrogenase
Fd: ferredoxin
Fdh: formate dehydrogenase
FNOR: flavin adenine dinucleotide-dependent NAD(P) oxidoreductase
HO-1: haem oxygenase 1
HO-2: haem oxygenase 2
HO: heme oxygenase
KO: KEGG orthology
MAG: metagenome-assembled genome
pCODH: putative nickel-containing CO dehydrogenase
PEP: phosphoenolpyruvate
PEPCK: PEP carboxykinase
PFL: pyruvate formate-lyase
PFOR: pyruvate: Fd oxidoreductase
RPKM: reads per kilobase of transcript per million reads mapped
WLP: Wood–Ljungdahl pathway
