# Supplementary material for: Phylogenetic diversity of putative nickel-containing carbon monoxide dehydrogenase-encoding prokaryotes in the human gut microbiome
Source: Microb Genom. 2024 Aug 21;10(8):001285. doi: 10.1099/mgen.0.001285 (PMC11338639; doi:10.1099/mgen.0.001285)
Supplement: Uncited Fig. S1. [file mgen-10-01285-s001.pdf]

Phylogenetic diversity of nickel-containing carbon monoxide dehydrogenase bearing prokaryotes in the human gut microbiome

Supplementary Figures

Yuka Adachi Katayama, Ryoma Kamikawa, Takashi Yoshida
Graduate School of Agriculture, Kyoto University

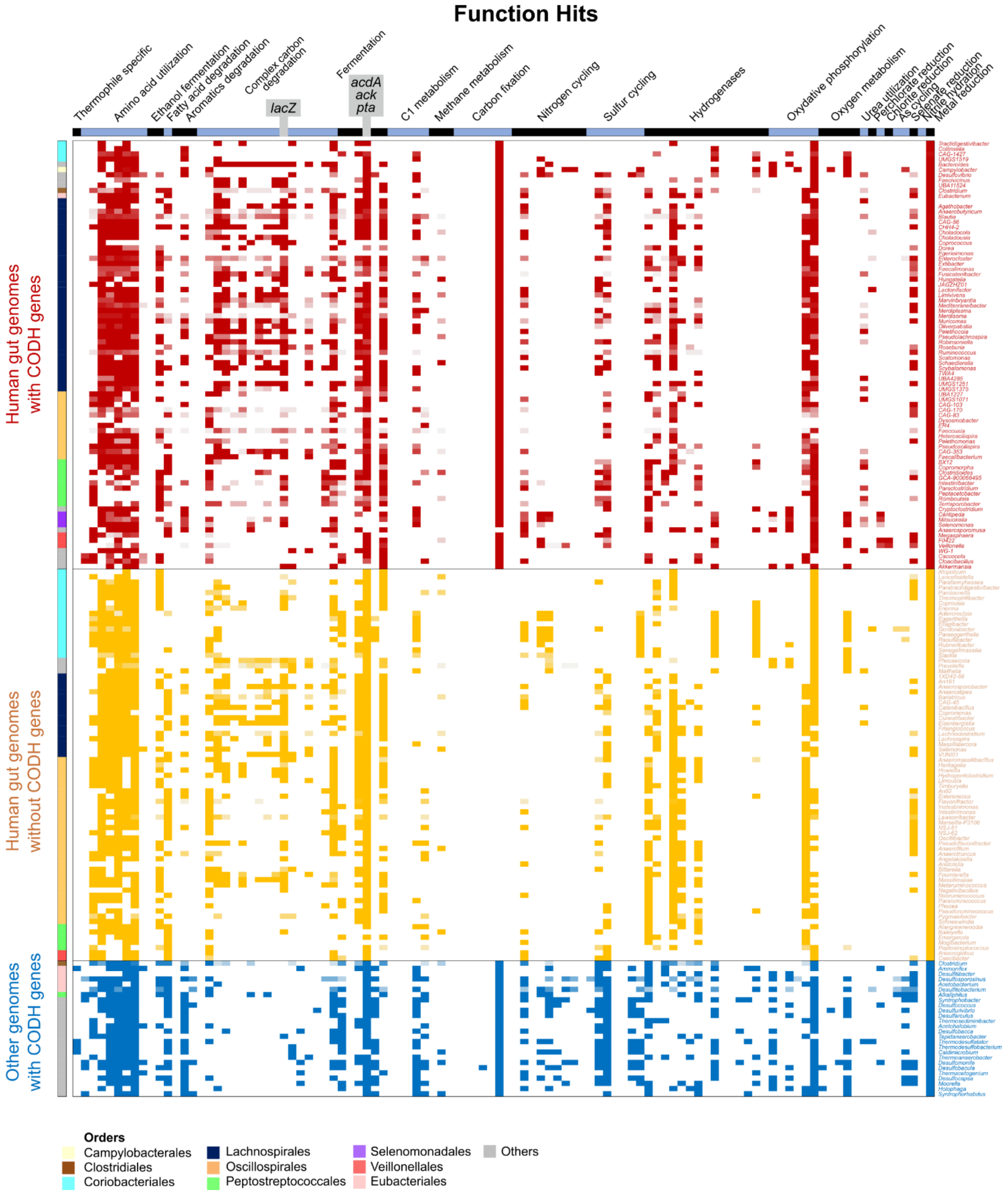

Fig. S1 Metabolic function hits detected in the three groups of the genomes

The metabolic functions of the three genome groups were analyzed using METABOLIC v4.0. The y-axis represents the genomes grouped by phylogeny (genera and orders) and origin. The first group consists of putative Ni-CODH (pCODH)-bearing genomes detected in the HumGut database (red). The second group comprises non-pCODH-bearing genomes, which belong to the same family as the pCODH-bearing genomes in the HumGut database (yellow). The third group comprised CO utilizers found in environments other than that of the intestines (blue). The x-axis represents the functions identified in each genome group. The functions were characterized by the presence of genes in METABOLIC. The following list represents the functions and the detected genes associated with each function, which are ordered from left to right in the columns: thermophilic-specific (reverse gyrase), amino acid utilization (4-aminobutyrate aminotransferase and related aminotransferases, aminotransferase class I and II, phosphoserine aminotransferase, ornithine/acetylornithine aminotransferase, branched-chain amino acid aminotransferase/4-amino-4-deoxychorismate lyase, aspartate/tyrosine/aromatic aminotransferase, histidinol-phosphate/aromatic aminotransferase, serine-pyruvate aminotransferase/archaeal aspartate aminotransferase), ethanol fermentation (aldehyde dehydrogenase, alcohol dehydrogenase), aromatics degradation (*catA*, *ubiX*||*bsdC*, *bcrABCD*), complex carbon degradation (cellobiosidase, cellulase, beta-glucosidase, arabinosidase, beta-glucuronidase, alpha-L-rhamnosidase, mannan endo-1,4-beta-mannosidase, alpha-D-xyloside xylohydrolase, beta-xylosidase, beta-mannosidase, beta-galactosidase, alpha-amylase, glucoamylase, pullulanase, isoamylase, chitinase, hexosaminidase), fermentation (*porA*, *adh*, *ldh*, *acdA*||*ack*||*pta*, *acs*, *pflD*), C1 metabolism (*mxaF* or *mdh*, *mauAB*, *fdhA*||*fghA*||*frmA*||mycoS_dep_FDH||*fae*, *fdoG*||*fdwB*||*fdoH*||*fdhAB*, *coxS*||*coxM*||*coxL*), methane metabolism (*pmoABC*, *mmoBD*, *mcrABC*), carbon fixation (Form I, Form II, *mcr*||K14469, K14466||K18861, K18861||4hbl, *cdhD*||*cdhE*||*cooS*, *aclAB*), nitrogen cycling (*amoABC*, *anfDKG*||*nifDK*||*vnfDKG*||*nifH*, *nxrAB*, *napAB*||*narGH*, *nrfADH*||*nirBD*, *nirKS*||*octR*, *norBC*, *nosDZ*, *hzoA*||*hzsA*), sulfur cycling (*fccB*||*sqr*, *dsrABD*||*asrABC*, *sdo*||*sor*, *sreABC*||*sor*, *soxBCY*, *aprA*||*sat*, *phsA*), hydrogenase (FeFe-group-a13, FeFe-group-a2, FeFe-group-a4, FeFe-group-b, FeFe-group-c1, FeFe-group-c2, FeFe-group-c3, Fe hydrogenase, Nife-group-1, NiFe-group-2ade, NiFe-group-2bc, NiFe-group-3abd, NiFe-group-3c, NiFe-group-4a-g, NiFe-group-4hi), oxidative phosphorylation (*nuoABC*, *ndhABC*, *sdhCD*, *petAB*||*fbcH*, *atpAB* (V/A-type), *atpAD* (F-type)), oxygen metabolism (*coxAB*, *ccoNOP*, *cyoABCD*, *cydAB*, *qoxAB*), urea utilization (*ureABC*), halogenated compound utilization (E3.8.1.2||*pcpC*||*cprA*||*pceA*), perchlorate reduction (*pcrAB*), chlorite reduction (*cld*), arsenic cycling (*arxA*||*aioA*, *arrA*), selenate reduction (*ygfMK*||*xdhD*), nitrile hydration (*nthAB*), and metal reduction (iron reduction series genes). Only *acdA*||*ack*||*pta* for fermentation and *lacZ* for complex carbon degradation are highlighted.

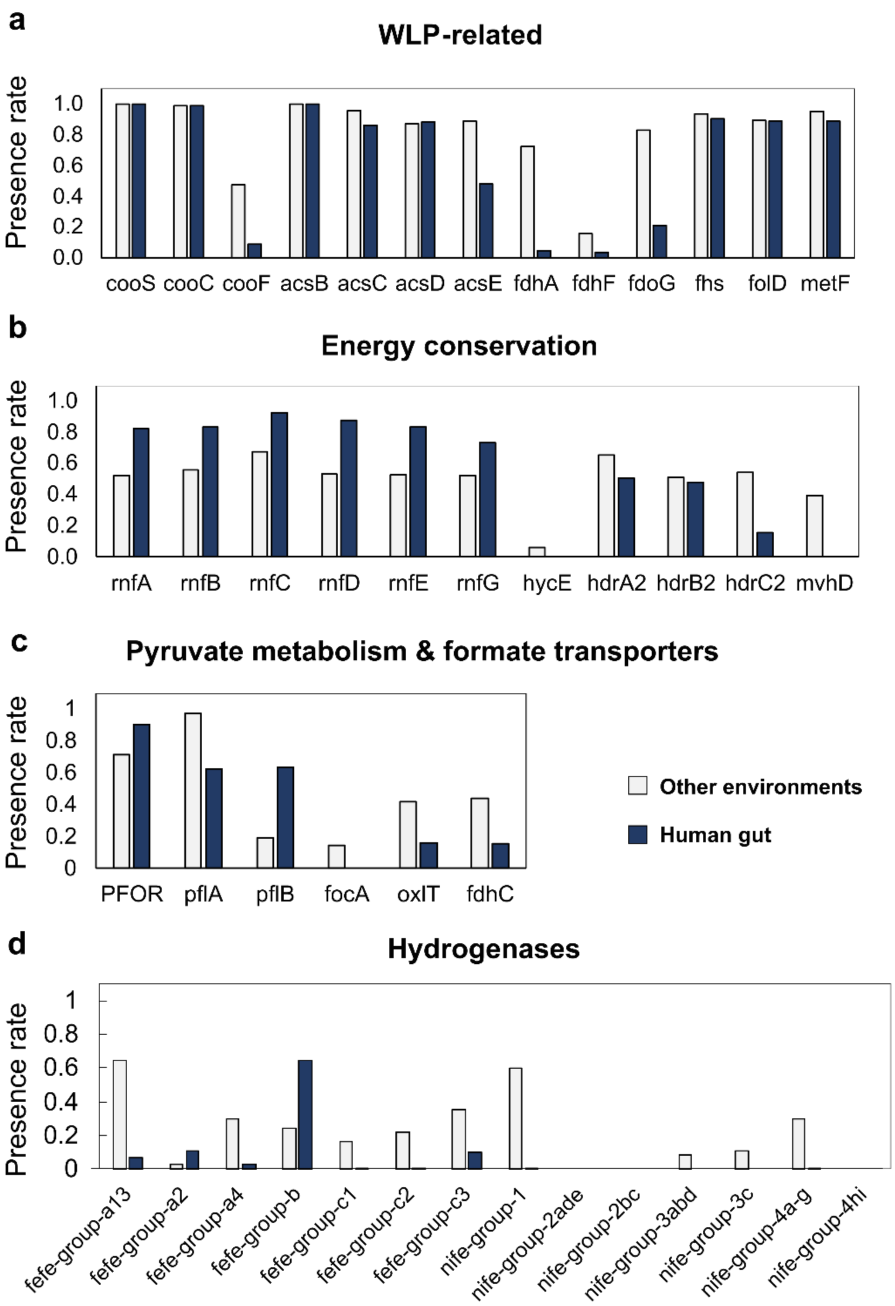

Fig. S2 Comparisons of the gene presence rates between human gut and other environmental putative Ni-CODH/ACS-containing bacteria.

The presence of WLP-related genes was compared between the genomes 667 of human gut bacteria and 554 bacteria from other environments, all of which possess putative Ni-CODH/ACS genes. The genes were classified into four categories: (a) WLP-related genes, (b) genes involved in energy conservation, (c) genes involved in pyruvate metabolism, and (d) hydrogenase genes. The presence of these genes was determined by analyzing KO annotations using eggNOG-mapper v2.2.5. The presence of hydrogenase genes was assessed using METABOLIC v4.0. *cooS*, carbon monoxide dehydrogenase catalytic subunit gene; *cooC*, carbon monoxide dehydrogenase maturation gene; *cooF*, ferredoxin-like protein-coding gene; *acsBCDE*, acetyl-CoA synthase gene; *fdhA*/*fdhF*/*fdoG*, formate dehydrogenase catalytic subunit gene; *fhs*, formyltetrahydrofolate synthase gene; *folD*, methylene-tetrahydrofolate dehydrogenase/cyclohydrolase gene; *metF*, methylene tetrahydrofolate reductase gene; *rnf*, ferredoxin:NAD^+ oxidoreductase gene; *hycE*, energy-converting hydrogenase catalytic subunit gene; *hdr-mvh,* methylenetetrahydrofolate reductase gene; *porABCD*, pyruvate ferredoxin oxidoreductase gene; *pfl*, pyruvate: formate lyase gene; *focA*/*oxlT*/*fdhC*, genes involved in formate transport. Other genes, genes involved in formate consumption and metabolism.

a **Raw data (genome count)**

fdhA vs group A13 FeFe hydrogenase		
	fefe-group-a13 +	fefe-group-a13 -
fdhA+	19	13
fdhA-	26	609

fdhA vs group A2 FeFe hydrogenase		
	fefe-group-a2 +	fefe-group-a2 -
fdhA+	1	31
fdhA-	69	566

fdhA vs group A4 FeFe hydrogenase		
	fefe-group-a4 +	fefe-group-a4 -
fdhA+	21	11
fdhA-	0	635

fdhA vs group B FeFe hydrogenase		
	fefe-group-b +	fefe-group-b -
fdhA+	21	11
fdhA-	407	228

fdhA vs group C1 FeFe hydrogenase		
	fefe-group-c1 +	fefe-group-c1 -
fdhA+	3	29
fdhA-	2	633

fdhA vs group C2 FeFe hydrogenase		
	fefe-group-c2 +	fefe-group-c2 -
fdhA+	0	32
fdhA-	2	633

fdhA vs group C3 FeFe hydrogenase		
	fefe-group-c3 +	fefe-group-c3 -
fdhA+	13	19
fdhA-	53	582

*fdhA = K05299

fdhF vs group A13 FeFe hydrogenase		
	fefe-group-a13 +	fefe-group-a13 -
fdhF+	15	9
fdhF-	30	613

fdhF vs group A2 FeFe hydrogenase		
	fefe-group-a2 +	fefe-group-a2 -
fdhF+	1	23
fdhF-	69	574

fdhF vs group A4 FeFe hydrogenase		
	fefe-group-a4 +	fefe-group-a4 -
fdhF+	19	5
fdhF-	2	641

fdhF vs group B FeFe hydrogenase		
	fefe-group-b +	fefe-group-b -
fdhF+	18	6
fdhF-	410	233

fdhF vs group C1 FeFe hydrogenase		
	fefe-group-c1 +	fefe-group-c1 -
fdhF+	3	21
fdhF-	2	641

fdhF vs group C2 FeFe hydrogenase		
	fefe-group-c2 +	fefe-group-c2 -
fdhF+	0	24
fdhF-	2	641

fdhF vs group C3 FeFe hydrogenase		
	fefe-group-c3 +	fefe-group-c3 -
fdhF+	7	17
fdhF-	59	584

*fdhF = K22015

fdoG vs group A13 FeFe hydrogenase		
	fefe-group-a13 +	fefe-group-a13 -
fdoG+	23	118
fdoG-	22	504

fdoG vs group A2 FeFe hydrogenase		
	fefe-group-a2 +	fefe-group-a2 -
fdoG+	9	132
fdoG-	61	465

fdoG vs group A4 FeFe hydrogenase		
	fefe-group-a4 +	fefe-group-a4 -
fdoG+	21	120
fdoG-	0	526

fdoG vs group B FeFe hydrogenase		
	fefe-group-b +	fefe-group-b -
fdoG+	66	75
fdoG-	362	164

fdoG vs group C1 FeFe hydrogenase		
	fefe-group-c1 +	fefe-group-c1 -
fdoG+	5	136
fdoG-	0	526

fdoG vs group C2 FeFe hydrogenase		
	fefe-group-c2 +	fefe-group-c2 -
fdoG+	0	141
fdoG-	2	524

fdoG vs group C3 FeFe hydrogenase		
	fefe-group-c3 +	fefe-group-c3 -
fdoG+	60	81
fdoG-	6	520

*fdoG = K00123

b **Odds ratio** **Red character: significantly different

fdhA vs group A13 FeFe hydrogenase	
Adjusted odds ratio	33.2
Upper confidence interval	73.6
Lower confidence interval	15.0

fdhA vs group A2 FeFe hydrogenase	
Adjusted odds ratio	0.4
Upper confidence interval	2.0
Lower confidence interval	0.1

fdhA vs group A4 FeFe hydrogenase	
Adjusted odds ratio	2376.2
Upper confidence interval	41654.9
Lower confidence interval	135.6

fdhA vs group B FeFe hydrogenase	
Adjusted odds ratio	1.0
Upper confidence interval	2.2
Lower confidence interval	0.5

fdhA vs group C1 FeFe hydrogenase	
Adjusted odds ratio	30.1
Upper confidence interval	158.8
Lower confidence interval	5.7

fdhA vs group C2 FeFe hydrogenase	
Adjusted odds ratio	3.9
Upper confidence interval	82.9
Lower confidence interval	0.2

fdhA vs group C3 FeFe hydrogenase	
Adjusted odds ratio	7.5
Upper confidence interval	15.9
Lower confidence interval	3.6

fdhF vs group A13 FeFe hydrogenase	
Adjusted odds ratio	32.8
Upper confidence interval	79.6
Lower confidence interval	13.5

fdhF vs group A2 FeFe hydrogenase	
Adjusted odds ratio	0.5
Upper confidence interval	2.8
Lower confidence interval	0.1

fdhF vs group A4 FeFe hydrogenase	
Adjusted odds ratio	909.8
Upper confidence interval	4335.7
Lower confidence interval	190.9

fdhF vs group B FeFe hydrogenase	
Adjusted odds ratio	1.6
Upper confidence interval	4.0
Lower confidence interval	0.7

fdhF vs group C1 FeFe hydrogenase	
Adjusted odds ratio	41.8
Upper confidence interval	223.9
Lower confidence interval	7.8

fdhF vs group C2 FeFe hydrogenase	
Adjusted odds ratio	5.2
Upper confidence interval	112.0
Lower confidence interval	0.2

fdhF vs group C3 FeFe hydrogenase	
Adjusted odds ratio	4.2
Upper confidence interval	10.3
Lower confidence interval	1.7

fdoG vs group A13 FeFe hydrogenase	
Adjusted odds ratio	4.4
Upper confidence interval	8.2
Lower confidence interval	2.4

fdoG vs group A2 FeFe hydrogenase	
Adjusted odds ratio	0.5
Upper confidence interval	1.1
Lower confidence interval	0.3

fdoG vs group A4 FeFe hydrogenase	
Adjusted odds ratio	187.9
Upper confidence interval	3123.4
Lower confidence interval	11.3

fdoG vs group B FeFe hydrogenase	
Adjusted odds ratio	0.4
Upper confidence interval	0.6
Lower confidence interval	0.3

fdoG vs group C1 FeFe hydrogenase	
Adjusted odds ratio	42.4
Upper confidence interval	772.0
Lower confidence interval	2.3

fdoG vs group C2 FeFe hydrogenase	
Adjusted odds ratio	0.7
Upper confidence interval	15.5
Lower confidence interval	0.0

fdoG vs group C3 FeFe hydrogenase	
Adjusted odds ratio	59.4
Upper confidence interval	138.0
Lower confidence interval	25.6

Fig. S3 The co-occurrence of genes for Fdh and FeFe hydrogenase in the genomes of putative CODH/ACS-bearing bacteria in the human gut

(a) The count of genomes containing homologue of Fdh genes and FeFe hydrogenase genes, as well as those lacking either gene. These counts were utilized to calculate odds ratio. (b) The odds ratio for the co-presence of putative Fdh and FeFe hydrogenase genes. The red characters indicate there were significant differences.

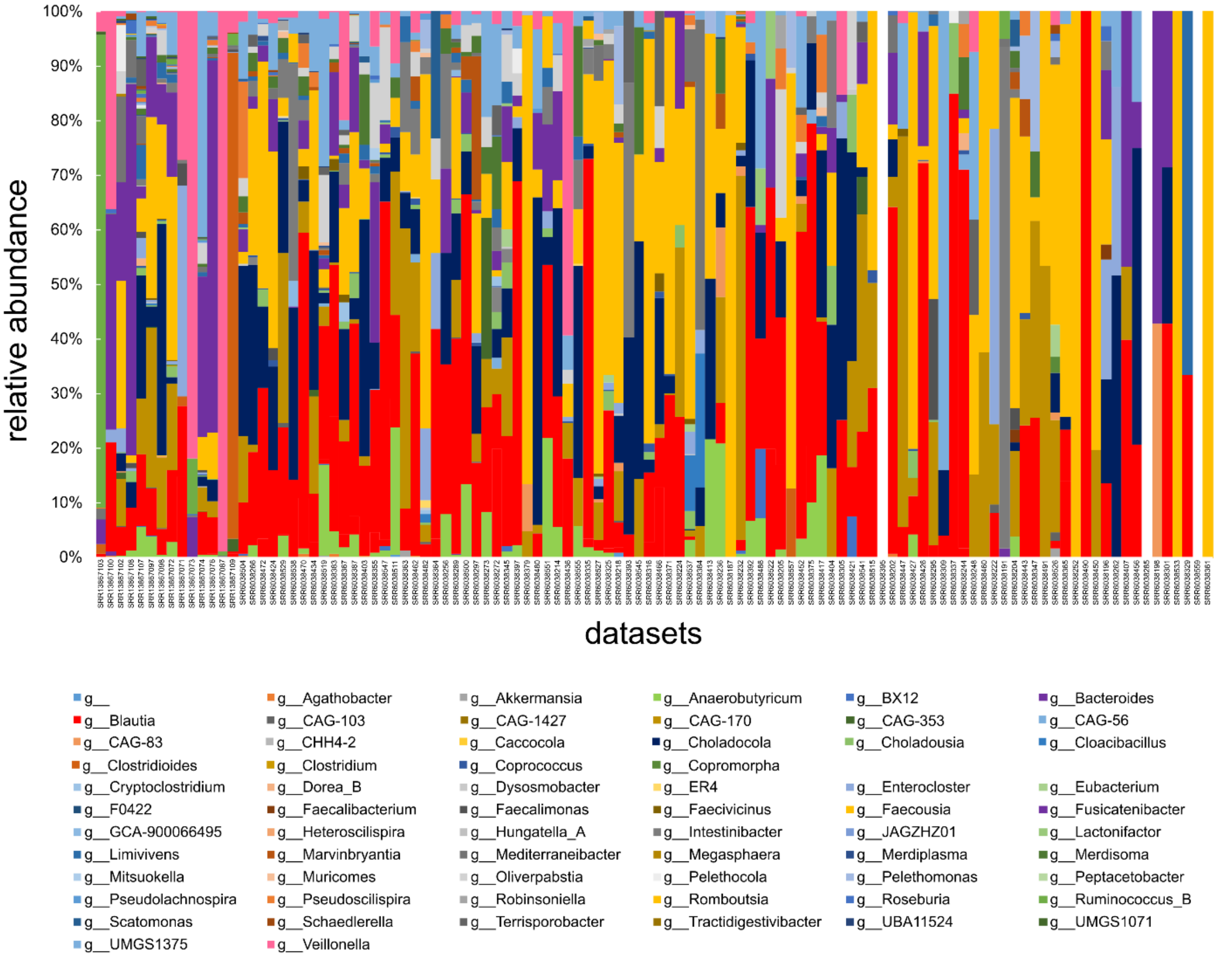

Fig. S4 Genera level diversity of putative Ni-CODH-bearing microbes in the healthy human gut metatranscriptomic datasets.

The genera level classification of the hosts of detected putative Ni-CODH transcripts are displayed. The y-axis represents the relative abundance of putative Ni-CODH transcripts. In the x-axis of the graph, datasets with a greater number of filtered RNA are aligned from left to right.
